# BoltzGen: Toward Universal Binder Design

**DOI:** 10.1101/2025.11.20.689494

**Published:** 2025-11-24

**Authors:** Hannes Stark, Felix Faltings, MinGyu Choi, Yuxin Xie, Eunsu Hur, Timothy O’Donnell, Anton Bushuiev, Talip Uçar, Saro Passaro, Weian Mao, Mateo Reveiz, Roman Bushuiev, Tomáš Pluskal, Josef Sivic, Karsten Kreis, Arash Vahdat, Shamayeeta Ray, Jonathan T. Goldstein, Andrew Savinov, Jacob A. Hambalek, Anshika Gupta, Diego A. Taquiri-Diaz, Yaotian Zhang, A. Katherine Hatstat, Angelika Arada, Nam Hyeong Kim, Ethel Tackie-Yarboi, Dylan Boselli, Lee Schnaider, Chang C. Liu, Gene-Wei Li, Denes Hnisz, David M. Sabatini, William F. DeGrado, Jeremy Wohlwend, Gabriele Corso, Regina Barzilay, Tommi Jaakkola

**Affiliations:** 1 MIT,; 2 Boltz,; 3 Open Athena,; 4 CTU Prague,; 5 IOCB Prague,; 6 NVIDIA,; 7 IOCB Boston,; 8 UC Irvine,; 9 MPI,; 10 UCSF,; 11 HHMI,; 12 Jameel Clinic

## Abstract

We introduce *BoltzGen*, an all-atom generative model for designing proteins and peptides across all modalities to bind a wide range of biomolecular targets. BoltzGen builds strong structural reasoning capabilities about target-binder interactions into its generative design process. This is achieved by unifying design and structure prediction, resulting in a single model that also reaches state-of-the-art folding performance. BoltzGen’s generation process can be controlled with a flexible design specification language over covalent bonds, structure constraints, binding sites, and more. We experimentally validate these capabilities in a total of eight diverse wetlab design campaigns with functional and affinity readouts across 26 targets. The experiments span binder modalities from nanobodies to disulfide-bonded peptides and include targets ranging from disordered proteins to small molecules. For instance, we test 15 nanobody and protein binder designs against each of nine novel targets with low similarity to any protein with a known bound structure. For both binder modalities, this yields nanomolar binders for 66% of targets. We release model weights, data, and both inference and training code at: https://github.com/HannesStark/boltzgen.

## 1 Introduction

De-novo binder design offers considerable potential for automating drug discovery. A number of previous techniques have been proposed to address parts of this challenge, including [Watson et al., 2023, Pacesa et al., 2024, Bennett et al., 2025, Mille-Fragoso et al., 2025]. Several key limitations remain, however. For example, many of the techniques are tailored to specific classes of biomolecules such as nanobodies or peptides. As models learn to emulate physics primarily through examples provided, we believe expanding the generality of the method further improves its design capabilities for specific classes as well. Another key limitation has to do with evaluation as methods are often tested on targets that have closely related complexes in the training data. The potential of de-novo binder design comes precisely from its presumed ability to extrapolate beyond easy targets. We believe design methods should be tested accordingly. Moreover, in real-world discovery campaigns, a number of additional requirements and constraints govern successful designs. It is important to be able to control the design process in a flexible manner.

**Figure 1:**
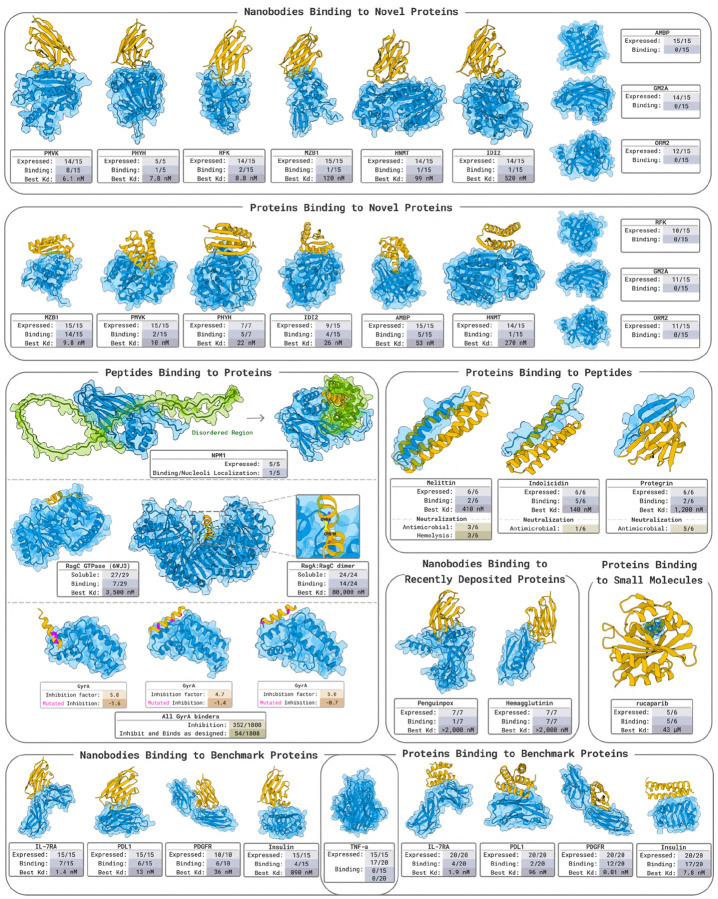
BoltzGen Wetlab Validation. We validate BoltzGen in collaboration with leading wet labs working on high-impact biological problems. These independently test designs for their specific applications. Additionally, we validate on 9 “novel targets” meaning that there are no proteins in a bound context with more than 30% sequence identity in the entire PDB.

Here we introduce BoltzGen, a binder design algorithm that addresses the above desiderata. At its core, the BoltzGen pipeline uses a single, all-atom generative model that unifies design and structure prediction. A purely geometry-based representation of designed residue types enables scalable training on both tasks simultaneously. As a result, unlike any previous design model, BoltzGen matches the performance of state-of-the-art folding models (Figure 20). BoltzGen’s structure-based reasoning about target-binder interactions supports design of high-affinity binders to novel targets, unrelated to complexes seen during training (Figure 1). We also provide a design specification language that allows for constraining binders across a variety of possible requirements, such as selecting a desired binding site or a (partial) structure for the target and covalent bonds or residue identity constraints in the design. The method is described in detail in Section 3.

### Wetlab Validation.

We experimentally validate our designs in a large-scale distributed effort involving multiple wetlabs. Each group selected targets and output modalities relevant to their specific application and then independently validated BoltzGen designs. To measure generalization capacity, we explicitly focus on targets that are dissimilar to any proteins for which bound structures exist.

Here we report the experimental results available to date; additional validation is ongoing. Some data are temporarily confidential at collaborators’ request, and we will update this work as further results become available.

1. Section 2.2: We design nanobodies against 9 novel targets for which there are no proteins with >30% sequence identity in a bound context in the entire PDB. Experimentally validating 15 or fewer designs against each target yields nM binders for 66% of them. The analogous experiment for protein binder designs results in the same success rate of nM binders against 66% of targets.
2. Section 2.3: When designing proteins to bind 3 bioactive peptides with diverse structures, we obtain nM binders for 2 and μM binders for the other, while only testing 6 binders per target.
3. Section 2.4: We generate and test 5 designs for binding the disordered region of NPM1 and obtain evidence of de-novo designs binding disordered proteins in live cells.
4. Section 2.5: When designing linear peptides to bind RagC, we obtain 7 binders after testing 29, with the highest affinity being 3.5μM.
5. Section 2.6: Similarly, designing disulfide-bonded peptides to bind the RagA:RagC dimer yields binders for 14 of 28 tested designs.
6. Section 2.7: We find hits when testing 7 nanobody designs against each of 2 novel targets in a yeast display assay.
7. Section 2.8: We obtain weak binders against two small molecules.
8. Section 2.9: Our campaign to design antimicrobial peptides binding to GyrA results in 19.5% of designs inhibiting cell growth by more than 4×.
9. Section 2.10: When testing at most 20 designed proteins and 15 nanobodies against 5 benchmark targets, we obtain nM binders against 80% of them with both modalities.

### Open Source Release.

We release training code, inference code, model weights, and all designs under the MIT License: https://github.com/HannesStark/boltzgen. The design pipeline is freely available, with easy-to-use interface to specify a binder-design problem and run BoltzGen, producing a filtered, ranked, diversity-optimized set of designs ready for experimental validation. We hope that fully open-sourcing the project puts state-of-the-art biomolecular design capabilities in the hands of any researcher and enables the community to build upon BoltzGen or contribute to BoltzGen-2.

## 2 Wetlab Results Summary

This section provides a summary of the wetlab results. Each subsection contains a figure that illustrates the best designs against each target. For the same set of experiments, more detailed descriptions are in Section 4, and their wetlab methodology is laid out in Appendix E. Unless mentioned otherwise, we provide the structure of the targets as input to BoltzGen.

### 2.1 Interpreting Affinities and Expression Numbers

#### Expression.

To test a designed binder, one typically first produces the DNA that encodes the design. If the DNA is introduced into an environment with molecular machinery that translates DNA into proteins, the protein is produced and the design is being *expressed*. Expression can fail for various reasons. For instance, a protein could fail to fold as intended, or a design could contain a large hydrophobic patch that binds to itself, causing aggregation (the proteins “clump up”). Usually, more stable and more soluble proteins have a higher chance of expressing well.

#### Affinity.

Binding affinity describes how tightly two molecules stick to each other. It is often quantified via their dissociation constant $K_{d}$, defined as the binder’s *off-rate* (how often they fall apart) divided by its *on-rate* (how often they come together). A smaller $K_{d}$ indicates that the molecules stay bound longer and interact more strongly. Natural protein–protein interactions cover a broad range of affinities: transient signaling complexes typically bind in the μM range, whereas stable complexes such as enzyme–inhibitor pairs or antibody–antigen assemblies can reach nM affinities. In contrast, therapeutic binders, such as monoclonal antibodies, engineered nanobodies, and peptide drugs, are often optimized to achieve tighter binding. Antibodies and nanobodies frequently reach picomolar or low-nanomolar affinities, while therapeutic peptides typically bind in the nanomolar range, depending on their size and conformational rigidity. Representative affinities for selected therapeutic binders are summarized in Table 1. Importantly, a high affinity is only the first step toward an effective therapeutic. It indicates that a molecule can recognize and stably engage its target, but not whether it will reach the target in the body, remain stable, avoid off-target interactions, or produce the desired biological effect.

### 2.2 Designing Nanobodies and Proteins against 9 Novel Targets

Experiments carried out by Adaptyv Bio.

#### Targets.

The majority of prior experimental validation of binder design models is carried out on targets that appear in their training data in complex with existing binders. In contrast to this, we choose 9 targets that are dissimilar to any other protein in PDB with an existing binder. For all 9 targets, we enforce that they are monomers and that there is no protein appearing in a bound structure in PDB with a sequence identity greater than 30%. Thus, it is possible that some of the targets do not even have a surface patch that allows for high-affinity protein-protein or nanobody-protein binding. We detail the potential therapeutic relevance of the targets in Section 4.1.

We evaluate BoltzGen’s ability to design both nanobodies and general proteins against these targets. Designing high-affinity nanobodies is generally more challenging, as it involves additional structural constraints that restrict the diversity of sequences and structures the model can generate. Nevertheless, nanobodies are often preferred as therapeutic modalities due to their favorable developability characteristics, including high solubility, robust expression yields, low aggregation propensity, good thermal and chemical stability, and ease of engineering [Jovčevska and Muyldermans, 2020].

#### Nanobody Designs.

We use BoltzGen to generate 60 000 nanobodies against each of the 9 targets *without* specifying any binding site. BoltzGen randomly samples from its 4 default nanobody scaffolds for each design (see Figure 9). Given a selected scaffold, we fix the structure and sequence of the framework region, but replace the 3 CDR regions with loops of random length.

#### Protein Designs.

We use BoltzGen to generate 60 000 proteins of lengths 80–140 against each of the 9 targets *without* specifying any binding site.

#### Results.

**Figure 2:**
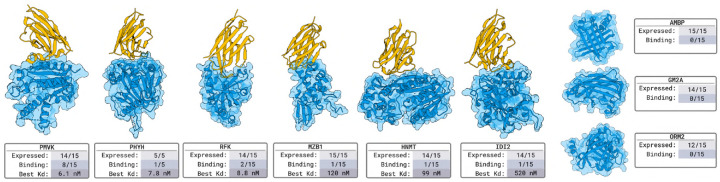
Nanobody binders for 9 novel targets.

**Figure 3:**
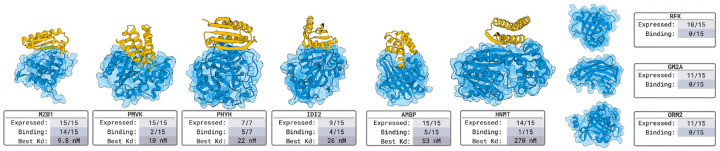
Protein binders for 9 novel targets.

Figures 2 and 3 report the per-target hit rates, top affinities, and expression yields. To evaluate binding specificity, we screened all successful designs against human serum albumin (HSA), a highly interactive off-target commonly used to detect nonspecific binding. No binder interacts with HSA.

#### Nanobody Design Results in Figure 2.

For each target, we select 15 nanobodies for experimental validation. Surface plasmon resonance (SPR) and biolayer interferometry (BLI) affinity assays confirm that we obtain nM-affinity binders for 6 out of 9 targets. This represents a 66% success rate against *novel* targets, none of which have similar proteins in a bound context in all of PDB.

#### Protein Design Results in Figure 3.

For each target, we evaluate a set of 15 designed protein binders. Using SPR and BLI, we detect nM binders for 6 out of 9 targets. These results represent a 66% success rate on *novel* targets without any similar protein in all of PDB that is in a bound structure.

### 2.3 Designing Proteins to Bind Bioactive Peptides with Diverse Structures

Experiments by A. Katherine Hatstat, Angelika Arada, Nam Hyeong Kim, Ethel Tackie-Yarboi, Dylan Boselli, Lee Schnaider, and William F. DeGrado.

#### Targets.

We design proteins to bind to three antimicrobial peptides and cytotoxic peptides as a class of biologically important compounds. We targeted multiple structural classes, including: 1) protegrin, a disulfide-rich beta hairpin; 2) melittin, which is intrinsically unfolded in dilute aqueous solution but forms a helix when bound to membranes; 3) indolicidin, which forms a polyproline II or amphipathic conformation in the presence of bilayers.


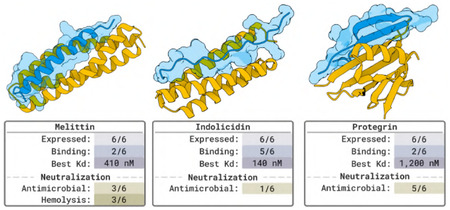


#### Designs and Results.

All selected designs were first screened for peptide binding in vitro via changes in intrinsic tryptophan fluorescence and/or by surface plasmon resonance (details in 4.2). As the target peptides are cytotoxic and antimicrobial peptides, we also assessed binders for their ability to neutralize antimicrobial activity and, where relevant, hemolysis. For each target, at least one binder design had single-digit μM affinity (for protegrin) or nM affinity (for indolicidin and melittin) and neutralized antimicrobial and, for melittin, hemolytic activity.

### 2.4 Designing Peptides to Bind the Disordered Region of NPM1.

Experiments by Yaotian Zhang, and Denes Hnisz.

#### Target.

The NPM1-c mutant of NPM1 is a known driver of Acute Myeloid Leukemia. We aimed to design peptides that bind to the disordered region of NPM1. NPM1 is particularly appealing as a target due to its intrinsic disorder and cellular localization: it naturally accumulates in the nucleoli, and peptides that bind to it are expected to co-localize there. Therefore, nucleolar localization of a designed peptide can be a proxy for assessing its binding to NPM1 in live cells.


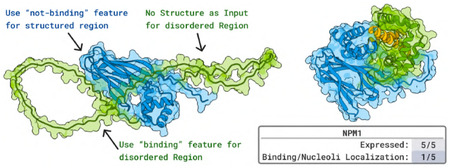


#### Designs.

We generate 20 000 peptide designs, each 40–80 residues in length, targeting the disordered region of NPM1. To guide the design process, we leverage BoltzGen’s binding site conditioning feature, explicitly directing the model to target the disordered region while avoiding interaction with the structured β-sheet region through its “not-binding” constraint. Additionally, we provide the structure of the ordered region and leave the disordered region flexible. Thus, tasking BoltzGen to model how the disordered region will fold and become structured in the presence of the designed peptide that is simultaneously being designed.

#### Results.

We experimentally test the top five highest-ranked designs in live human cells using fluorescence readouts based on GFP attached to the designs (see Section 4.3 for details). One design reliably localized to the nucleoli, suggesting successful binding to NPM1. Thus, we obtain a *de-novo* designed protein with *in vivo* evidence of binding to a disordered protein. Importantly, this in-cell assay provides insight beyond binding affinity. It also reflects functional viability, including the design’s selectivity for NPM1 over potential off-targets that do not localize to the nucleoli. However, this experiment does not definitely confirm that the binding occurs specifically at the disordered region - the main evidence for this comes from the BoltzGen-generated structure and structure predictions within the BoltzGen pipeline.

### 2.5 Designing Peptides to Bind a Specific Site of RagC GTPase

Experiments by Shamayeeta Ray, Jonathan T. Goldstein, and David M. Sabatini.

#### Target.

RagC GTPase is a central part of a cellular pathway for sensing nutrients and regulating cell growth, protein synthesis, and other metabolic processes. There are no existing peptide binders against RagC.


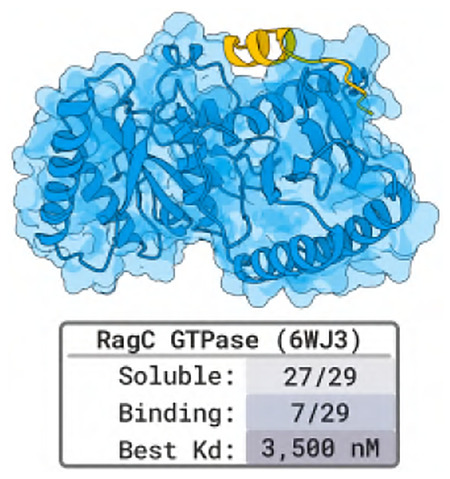


#### Designs and Results.

With one of RagC’s interaction surfaces as binding-site input for BoltzGen, we generate 10 000 ranked designs of length 5–20. We test 29 in a binding affinity assay (SPR), and find 7 binders. The highest affinity is 3.5μM and the second highest 60μM.

### 2.6 Designing Disulfide Bonded Cyclic Peptides to Bind a Specific Site of RagA:RagC

Experiments by Shamayeeta Ray, Jonathan T. Goldstein, and David M. Sabatini.

#### Target.

The RagA:RagC dimer is part of a cellular pathway responsible for sensing nutrients and regulating cell growth, protein synthesis, and other metabolic processes. There are no existing peptide binders against the RagA:RagC dimer.


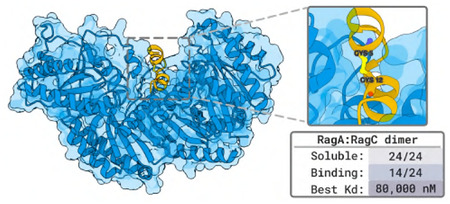


#### Designs.

We use BoltzGen to design 50 000 ranked disulfide-cyclized peptides of size 10–18 against the RagA:RagC dimer with one of its interaction surfaces specified as binding site. The aim of introducing a disulfide bond between two residues of the peptide is to stabilize it and reduce its flexibility (rigidity reduces entropy loss during binding, thus potentially aiding stronger binding). To achieve this with BoltzGen, we specify the design to contain two cysteines that are covalently bonded. The cysteines are separated by six designed residues, with an additional one to five designed residues flanking either side of this eight-residue segment.

#### Results.

We test 24 designs in a binding affinity assay and find 14 binders. For 8 of those, we resolved their affinities (SPR) and obtained 80μM as the highest 164μM as the second highest affinity.

### 2.7 Designing Nanobodies that Bind Penguinpox and Hemagglutinin

Experiments by Jacob A. Hambalek, Anshika Gupta, Diego Taquiri Diaz, and Chang C. Liu.

#### Targets.

We choose two monomer targets that were recently deposited in PDB. The first target is the cyclic GMP-AMP phosphodiesterase of Penguinpox (cGAMP PDE), a protein known to inhibit host STING signaling by degrading cyclic dinucleotides [Hobbs et al., 2024]. The second is Filamentous Hemagglutinin (FhaB), an adhesion protein expressed by the pathogen Bordetella, which allows the pathogen to colonize and infect hosts [Costello et al., 2025].


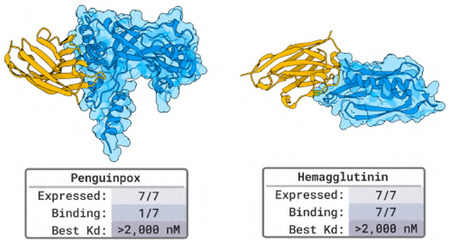


#### Designs and Results.

We generate 60 000 nanobodies against each target in the fashion described in Section 2.2 and select 7 per target for experimental characterization. A yeast surface display assay shows binding signal for a nanobody to bind Penguinpox and for 7 to bind Hemagglutinin. The assay does not allow for computing a binding affinity but indicates that it is at best 2μM. We note that we carried out similar experiments on a set of designs from a previous version of BoltzGen that suffered from a serious flaw resulting in close-to-random ranking and filtering (Details in D). For those designs no hits were found.

### 2.8 Designing Proteins that Bind to Small Molecules

Experiments by Nam Hyeong Kim and William F. DeGrado.

#### Targets.

We evaluate BoltzGen’s ability to design binders against two small molecules: rucaparib and a rhodamine derivative. Binders to these targets could serve as components in biosensors, delivery systems, or detoxification agents.


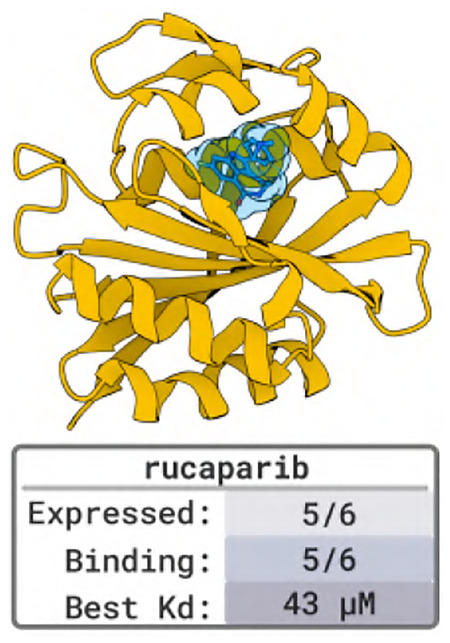


#### Designs and Results.

We generate 10 000 protein designs targeting rucaparib and 20 000 targeting the rhodamine derivative with design lengths ranging from 140 to 180 residues. We select six designs against rucaparib for experimental validation, five of which show binding with affinities between 50 and 150μM. For the rhodamine derivative, four designs were tested experimentally, all showing weak binding with affinities between 30 and 250μM.

Previous computational work [Lu et al., 2024] designed a low-nanomolar binder to rucaparib through a specialized, expert-guided approach that involved identifying specific chemical groups on the small molecule. In contrast, our work demonstrates that a general-purpose design model, BoltzGen, can generate diverse scaffolds with moderate binding affinity and simpler customization.

### 2.9 Designing Antimicrobial Peptides that Inhibit the GyrA to GyrA Interaction

Experiments by Andrew Savinov, and Gene-Wei Li.

#### Target.

We used BoltzGen to design inhibitory peptides of the essential bacterial protein DNA gyrase subunit A (GyrA), a target of interest for developing antibiotics. For its function, GyrA needs to interact with a copy of itself. When disrupting this interaction in bacteria, they die.


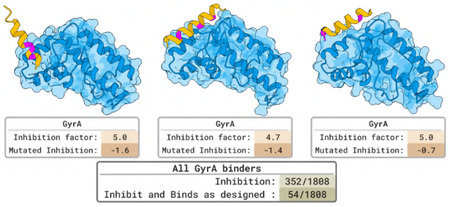


#### Designs and Results.

We specify the surface where GyrA interacts with a copy of itself as the binding site when generating peptides of length 10–50. We select 1 808 designs for experimental validation in a growth inhibition assay. Of these, 352 (19.5%) were found to inhibit *E. coli* growth by more than 4×. In a second experiment, we replace the design’s three closest residues to the target (pink in the Figure) with alanines to verify whether they bind as intended. 54 (3.0% of total) of the growth inhibitors lose their activity after introducing these mutations. The inhibitory effects of the 54 successful designs were, in most cases, strong enough to completely eliminate the cell populations in which they were expressed.

### 2.10 Designing Nanobodies and Proteins against 5 Benchmark Targets

Experiments carried out by Adaptyv Bio.

#### Targets.

We designed binders against PD-L1, TNFα, PDGFR, IL-7Rα, and InsulinR, which were considered in previous work [Chai-Discovery et al., 2025, Zambaldi et al., 2024]. All these targets have known binders that are included in the training data of most previous models and in our training data.

1. TNFα There are 18 matching PDB complexes, i.e. entries with more than 1 protein entity and with over 90% sequence identity to the structure used to design binders. See, for example, PDB 5M2I, released in 2017, showing TNFα in complex with picomolar-affinity nanobodies [Beirnaert et al., 2017].
2. **PD-L1** There are 37 matching PDB complexes. See, for example, PDB 5JDS, released in 2017, showing PD-L1 in complex with a 3.0-nanomolar-affinity nanobody [Zhang et al., 2017].
3. **PDGFR** The PDB entry 3MJG used for making designs, released in 2010, shows PDGFR in complex with its binding partner PDGF, and previous work has reported *de novo* mini protein binders with nanomolar affinities [Cao et al., 2022].
4. IL-7Rα There are 6 matching PDB complexes, such as PDB entry 6P50, released in 2019, which shows IL-7Rα in complex with a 1-nanomolar-affinity Fab [Hixon et al., 2020].
5. **InsulinR** There are 74 matching PDB complexes. The PDB entry 4ZXB used for design, released in 2016, shows the target in complex with four Fabs (83-7 and 83-14) [Croll et al., 2016].

#### Nanobody Designs.

We generate 60 000 nanobodies (except for TNFα, where we only generate 30 000) against each of the 5 targets while specifying the binding sites listed in Chai-Discovery et al. [2025]. BoltzGen randomly chooses between its 4 default scaffolds for each design (see Figure 9). For a selected scaffold, we fix the structure and sequence of the framework region, but replace the 3 CDR regions with loops of random length.

#### Protein Designs.

We use BoltzGen to generate 60 000 proteins (except for TNFα where we only generate 30 000) of lengths 80–120 against each of the 5 targets while specifying the binding sites listed in Chai-Discovery et al. [2025].

#### Nanobody Binder Results in Figure 4.

For each target, we evaluate a set of 15 or fewer designed protein binders. SPR and BLI assays confirm nM-affinity binders for 4 out of 5 targets (an 80% success rate).

#### Protein Binder Results in Figure 5.

We tested 20 designed protein binders per target. SPR and BLI assays identified binders for 4 out of 5 targets, including pM hits on PDGFR, yielding an 80% success rate. The missing target is TNFα for which previous work [Chai-Discovery et al., 2025] successfully designed against. To evaluate binding specificity, we screened all successful designs against human serum albumin (HSA), a highly interactive off-target commonly used to detect nonspecific binding. The only instance of off-target activity is a designed binder for IL-7Rα (27 nM affinity), which exhibits additional binding to HSA at 333 nM.

We note that these benchmark targets include cases where high-affinity binders are already present in public datasets. In such settings, a model may succeed by reusing or recombining interaction motifs encountered during training, rather than by generating truly novel binding solutions. Consequently, results on these benchmark targets provide marginal evidence toward a model’s ability to generalize to discovery scenarios involving targets without known binders.

## 3 Method

BoltzGen is a single all-atom diffusion model capable of performing both structure prediction and protein design. The model takes a set of molecular entities as input and outputs their all-atom three-dimensional structure. Molecular entities include small molecules, RNA, DNA, or protein sequences, along with any post-translational modifications and covalent bonds. New proteins are sampled by specifying *design residues*, for which the model generates both the all-atom structure and amino acid identities. Structure prediction and design capabilities can be exercised in tandem; for example, when generating a binder given only the sequence of the target, the model simultaneously folds the target and designs the binder’s atomic structure, producing a bound complex.

### Unified Design and Structure Prediction.

**Figure 7:**
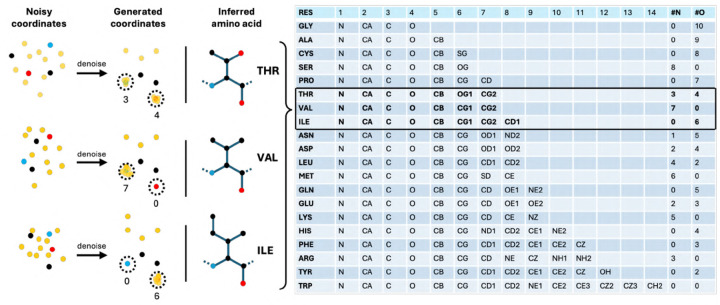
Residue Type Encoding In BoltzGen, each designed residue is represented using 14 atoms. To determine the residue type, the model is trained to superpose a subset of these atoms onto specific backbone atoms. These auxiliary atoms act as markers and are discarded after decoding. For example, placing three atoms on the backbone nitrogen and four on the backbone oxygen is interpreted as a threonine. The atoms in positions 5, 6, and 7 are then assigned as the threonine CB, OG1, and CG2 atoms, respectively.

The joint all-atom sequence and structure sampling ability of the model and its scalable training come from its purely geometry-based representation of designed amino-acid types. This representation encodes residue identities according to the position of the “left-over” atoms of side chains (see Figure 7). This change helps maintain a scalable architecture and its associated diffusion training process, similar to state-of-the-art biomolecular structure prediction methods.

### Design Specification Language.

Predictions can be conditioned on a broad set of additional inputs. These include standard annotations such as desired residue types and covalent bonds, as well as secondary structure identity, binding site location, and structure templates. The conditions can be incorporated with the help of a rich design specification language, allowing us to support the needs of our experimental collaborators. As a result, we can address a wide range of design challenges, including diverse modalities such as nanobodies, cyclic peptides with various cyclizations, helicons, or cyclotides.

In addition to the core generative model, we provide a comprehensive design pipeline that includes: (1) the initial generation of candidate designs, (2) optional sequence redesign through inverse folding, (3) evaluation of refolding quality and, for small molecule targets, affinity estimation, (4) ranking and filtering of designs, and (5) selection of a final candidate pool with diversity optimization.

### 3.1 All-atom Generative Model Formulation

#### Model Representations.

All non-designed molecular entities provided as input to the model are represented at the atomic level, including atom positions, element types, and charge. These atoms are grouped into tokens, such as residues for proteins or nucleotides for RNA and DNA. Designed residues, which do not have a specified amino acid type during generation, use a fixed-size representation consisting of exactly 14 atoms, some of which serve as *virtual* placeholders. Once the model determines the final residue types, these virtual atoms are discarded, as done in other approaches that employ a fixed number of residues to circumnavigate the issue that a residue’s atom count is unknown before its generation [Qu et al., 2025, Chu et al., 2024, Butcher et al., 2025].

#### Geometric Encoding of Residue Type.

Instead of generating a discrete residue label, the model encodes the residue identity through the geometry of the 14-atom representation (see Figure 7).

Specifically, it learns to place the virtual atoms on top of designated backbone atoms to signal the intended residue type. The first four atoms in each designed residue are fixed as the backbone N, Cα, C, and O atoms, in that order. As a result, residue types can be inferred directly from the generated structure by counting how many atoms are placed within 0.5 Å of each backbone atom. Any remaining atoms that are not superposed onto the backbone are interpreted as the side chain. For example, proline is encoded by placing 7 atoms on the backbone oxygen, while threonine is represented by placing 3 atoms on the nitrogen and 4 on the oxygen.

This geometric encoding allows the model to operate entirely in a continuous space, avoiding the need to mix discrete and continuous representations. As a result, it enables efficient joint training for both structure prediction and design tasks.

#### Diffusion Process.

Because the model operates on a continuous space, we can use the same diffusion process as Boltz-2 [Passaro et al., 2025]. The only difference is that the data samples now contain additional virtual atoms for the designed residues.

Formally, let $X\sim p_{\text{data}},X\in R^{N\times 3}$ the 3D atomic coordinates of a training sample and let $X_{t}$ follow the forward diffusion process

(1) $$dX_{t}=\sqrt{2t}dB_{t},$$

with initial condition $X_{0}\sim p_{\text{data}}$. For large $T,X_{T}$ will be approximately Gaussian with variance $T^{2}$. This process can be reversed to obtain samples from the data distribution starting from Gaussian noise. Reversing the diffusion process requires a denoiser $D_{\theta }(x,t;z)$, parameterized by learnable weights θ. The goal of the denoiser is to approximate the posterior mean

(2) $$D_{\theta }\left(x,t;z\right)\approx \mu_{t}\left(x\right)=E_{X_{0}|X_{t}=x}\left[X_{0}\right],$$

conditioned on trunk features z. The model is trained using a standard denoising loss

(3) $$ℒ\left(\theta \right)=E_{t}E_{X_{0},X_{t}}\left[w\left(t\right)\cdot {\left\|D_{\theta }\left(X_{t},t;z\right)-X_{0}\right\|}^{2}\right],$$

where w(t) is a weighting function. In the absence of parametric constraints, the minimizer of this loss is $\mu_{t}$, the posterior means.

### 3.2 Architecture

**Figure 8:**
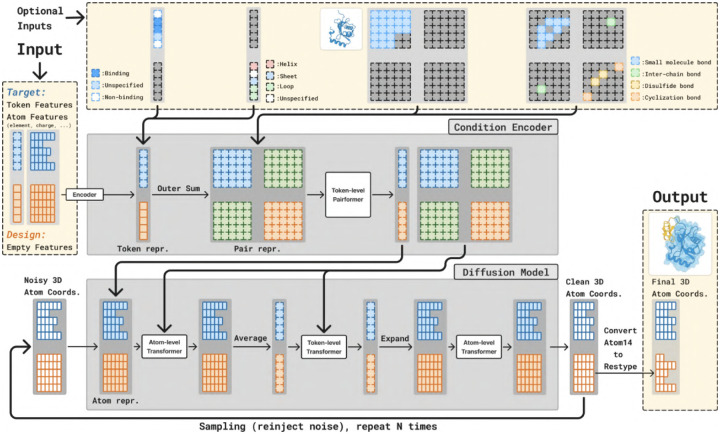
Model Architecture The architecture preserves the main components of the AlphaFold3 and Boltz-2 architectures, including the condition encoder (trunk) and diffusion model. The main difference lies in the inclusion of design tokens and additional inputs such as binding site and target structure.

The model preserves the Boltz-2 architecture with some modifications to include additional conditioning inputs, as shown in Fig. 8. The model is split into two parts. The larger *Trunk* produces token and pairwise representations used to condition the *Diffusion Module*, which generates the structure. The trunk is only run once, while the diffusion module is run many times to progressively denoise the 3D coordinates of all atoms.

#### Trunk.

The trunk operates on tokenized structures, where proteins are tokenized into amino acids, RNA and DNA into nucleotides, and small molecules into atoms. Each token consists of a group of atoms with associated features such as charge and element type, along with token-level attributes including residue index, amino acid type, and a flag indicating whether the residue is designed. All features are encoded into vector representations. Atom-level embeddings are averaged to produce token-level vectors, and a pair representation is constructed using an outer-sum of the token embeddings. Both token and pair representations are then passed through a PairFormer stack. Each PairFormer block includes triangle multiplicative and triangle attention layers that update the pair representations, along with a transformer layer that updates the token representations using the pair features as the attention bias.

#### Diffusion Module.

The diffusion module takes noisy 3D atomic coordinates as input and predicts denoised coordinates. It uses a standard transformer architecture that operates on both atom and token levels, consisting of 3 atom-level layers, followed by 24 token-level layers, and concluding with another 3 atom-level layers. The atom-level layers utilize sequence-local attention. Transitions between atom and token levels are handled by averaging or expanding the representations. Conditioning information from the trunk is incorporated by adding expanded token-level features to the atom-level input representations, and by biasing the attention layers based on the pair representation.

As in Boltz-2, AlphaFold3 and EDM [Karras et al., 2022], the diffusion module is preconditioned to parameterize the denoiser $D_{\theta }$,

(4) $$D_{\theta }\left(x,t;z\right)=\frac{\sigma_{\text{data}}^{2}}{\sigma_{\text{data}}^{2}+t^{2}}x+\frac{t\cdot \sigma_{\text{data}}}{\sqrt{\sigma_{\text{data}}^{2}+t^{2}}}F_{\theta }\left(\frac{x}{\sqrt{\sigma_{\text{data}}^{2}+t^{2}}},\frac{1}{4}\text{log}\left(t\right);z\right),$$

where $F_{\theta }$ is the diffusion module. This parameterization is derived in [Karras et al., 2022] so that (1) the inputs to the network $F_{\theta }$ have unit variance (2) the effective training target of $F_{\theta }$ has unit variance and (3) the scalar multiplier of $F_{\theta }$ in Eqn. 4 is minimal.

#### Controllability.

**Figure 9:**
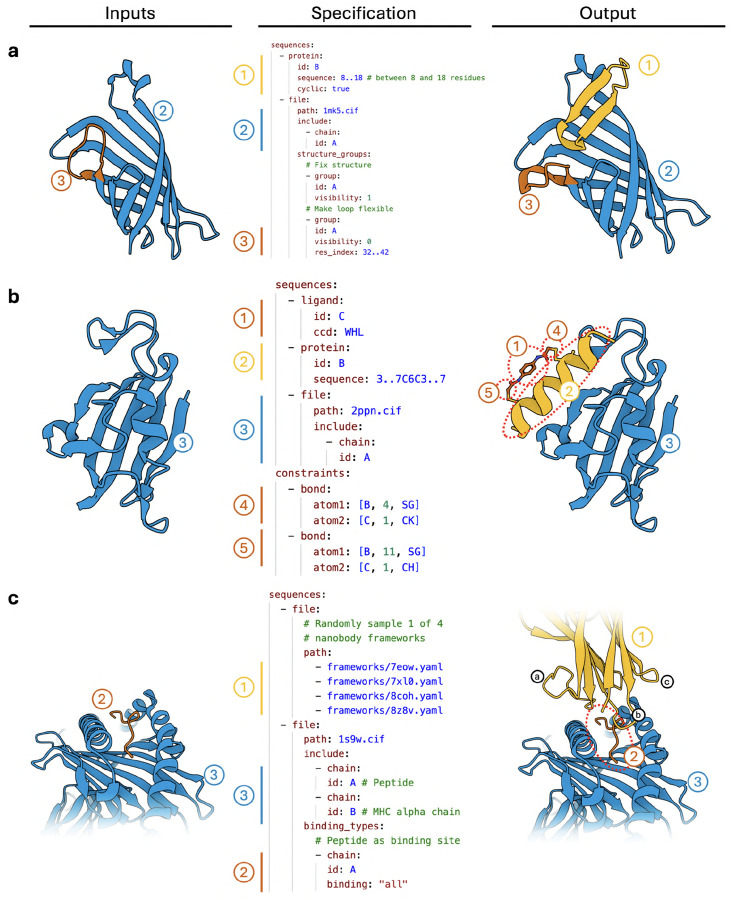
Design Specification Language. 3 examples of how the BoltzGen design specification can be used to solve different design tasks. (**a)** Designing a cyclic peptide against streptavidin. Part of the target structure is left flexible (3, orange) and is predicted to change conformation upon binding the design (1, yellow). (**b**) Designing a helicon binder. The helicon is created by including the staple molecule (1, WHL) and specifying covalent bonds between two cysteines in the design and the staple (4,5, orange). (**c**) Designing a nanobody against a peptide-MHC complex. The peptide (2, orange) is specified as a binding site, and the designed regions are limited to the 3 CDR loops (a,b,c) of the nanobody (1, yellow). The nanobody frameworks are themselves yaml files that specify the PDB structure to use and which are parts to design. With multiple specifications, a random one is sampled for each design.

Several additional inputs can optionally be provided to BoltzGen to steer the generation process according to user-specified requirements. Figure 8 shows where these inputs are integrated into the architecture, and Figure 9 illustrates the resulting expressive design specification language.

1. **Covalent Bonds.** Covalent bonds can be specified between individual atoms, in which case the identity of the residues that contain the bound atoms must be specified.
2. **Structure Conditioning.** Parts of the structure can be specified to the model via pairwise distances, for example to perform motif scaffolding. All structures are specified in *structure groups*, where all pairwise distances between atoms in the same structure group are fixed, but not across groups. For example, during nanobody design, the nanobody framework structure and the target structure can be fixed but their relative positions to each other can be left unconstrained by putting them in different structure groups.
3. **Binding Site.** Residues can be specified as binding, or not-binding. The model will try to place designed residues close to binding residues and away from non-binding residues.
4. **Secondary Structure.** Designed residues can also be specified to be part of alpha-helices, beta-sheets, or coils.

Covalent bonds, specified as a matrix of pairwise bonds, and pairwise distances for structure conditioning are both encoded and added to the trunk’s input pair representation. Binding and secondary structure labels are incorporated into the input token representation.

These conditioning options can be used to control the model and address a variety of design tasks. For example,

1. **Cyclic Peptides** can be designed by specifying a covalent bond. This includes disulfide-stapled peptides, head-to-tail cyclic peptides, and any other type of cyclization.
2. **Helicons** can be designed by including a staple molecule in the model’s input and enforcing covalent bonds between the staple and the sulfurs of two cysteines in the design.
3. **Nanobodies** can be designed by enforcing the design to adhere to a given template, allowing the model to generate the CDRs as well as place the scaffold in relation to the target.

Figure 9 also illustrates how to realize these examples using the BoltzGen design interface.

### 3.3 Training

**Figure 10:**
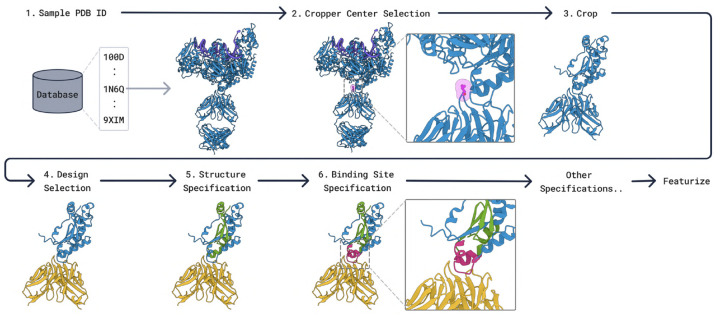
Crop, Selection, and Specification During training, we sample one PDB structure from the database and crop it using our cropping algorithm 4. On top of this cropped structure, we optionally select chains that will be designed (yellow). Diverse conditions, such as fixed target substructures (green), binding sites (red), and more, are also optionally specified. Cropped, selected, and specified structure is then featurized to be supplied to the model.

The model is trained with a diffusion objective with a mixture of experimental and self-distilled biomolecular structures. This data is then randomly cropped and employed in a diverse set of tasks by randomly selecting parts of the structure to be designed or conditioned on. The procedure is described in Figure 10. This conditioning sampling process, combined with the diffusion objective, supervises BoltzGen to simultaneously learn folding, binder design, motif scaffolding, and more, resulting in a universal binder design model that maximally extracts information from the data.

#### Training Data.

Our data pipeline mostly retains the datasets used in Boltz-2 [Passaro et al., 2025], while adapting the sampling procedure for the task of biomolecular design. Specifically, we use experimental structures from the Protein Data Bank (PDB) [Berman et al., 2000], as well as self-distilled structures from AlphaFold2 (AFDB), as well as Boltz-1 (for protein-ligand, RNA, and DNA-protein structures) (see Appendix B.1 for dataset details). Our data also differs from Boltz-2 by not including the upsampled antibody and TCR datasets, since including them reduces generation diversity.

#### Cropping.

The crop size for folding is up to 768 residues, as done in [Abramson et al., 2024, Passaro et al., 2025]. Crop size for generative tasks (binder design, motif scaffolding, and unconditional design) is reduced to 512 residues, to accommodate augmented fake atom representations (Appendix B.2).

#### Diffusion Objective.

The loss used to train the model is

(5) $$ℒ\left(\theta \right)=E_{\begin{array}{c} X\sim p_{\text{data}}\\ t\sim p_{\text{noise}}\\ \epsilon \sim 𝒩 \end{array}}\left[w\left(t\right)\left({ℒ}_{\text{MSE}}\left(\theta ;X,t,\epsilon \right)+{ℒ}_{\text{bond}}\left(\theta ;X,t,\epsilon \right)\right)+{ℒ}_{\text{smooth\_lDDT}}\left(\theta ;X,t,\epsilon \right)\right],$$

where $p_{\text{noise}}$ is described below, ϵ is standard isotropic Gaussian noise, $\sigma_{\text{data}}^{2}$ is the variance of the data, ${ℒ}_{\text{MSE}},{ℒ}_{\text{bond}}$, and ${ℒ}_{\text{smooth\_lDDT}}$ are the three components of the loss described below and the weighting is defined as

(6) $$w\left(t\right)=\frac{t^{2}+\sigma_{\text{data}}^{2}}{{\left(t\cdot \sigma_{\text{data}}\right)}^{2}}.$$

Let $\overset{ˆ}{X}=D_{\theta }(X+t\epsilon ,t;z)$ be the output of the denoiser. The MSE loss is a weighted version of the denoising loss including a rigid alignment of the denoiser output and target,

(7) $${ℒ}_{\text{MSE}}\left(\theta ;X,t,\epsilon \right)=\frac{1}{3}\sum_{l}w_{l}{\left\|{\overset{ˆ}{X}}_{l}-X_{l}^{\text{aligned}}\right\|}_{w}^{2},$$

where l iterates over atoms in the structure, $X^{\text{aligned}}$ is rigidly aligned to $\overset{ˆ}{X}$, and $w_{l}$ upweighs nucleotide and ligand atoms,

(8) $$w_{l}=\left\{\begin{array}{ll} 1 & \text{if protein}\\ 6 & \text{if DNA or RNA}.\\ 11 & \text{if ligand} \end{array}\right.$$

The bond loss encourages bond length correctness,

(9) $${ℒ}_{\text{bond}}\left(\theta ;X,t,\epsilon \right)=\frac{1}{\left|ℬ\right|}\sum_{l,m\in ℬ}{\left(\left\|{\overset{ˆ}{X}}_{l}-{\overset{ˆ}{X}}_{m}\right\|-\left\|X_{l}^{\text{aligned}}-X_{m}^{\text{aligned}}\right\|\right)}^{2},$$

Finally, ${ℒ}_{\text{smooth\_lDDT}}$ is described in [Abramson et al., 2024] and is a smooth version of the lDDT which can be directly optimized.

The noise sampling distribution $p_{\text{noise}}$ used during training is

(10) $$\sigma_{\text{data}}\cdot \text{exp}\left(-1.2+1.5\cdot 𝒩\left(0, 1\right)\right).$$

#### Training Tasks.

We train BoltzGen on a number of tasks (Appendix B.3 for more details) under the different forms of conditioning above to obtain a general and controllable algorithm. This not only results in generality but could also improve performance for individual tasks as the model is exercised on the data in more contexts, increasing the number of examples to extract generalizable patterns from and learn to emulate physics. Each task is distinguished by which parts of a cropped structure are chosen to be designed. These selected parts are represented using the fixed-size representation with virtual atoms described above, and their residue and atom types are masked.

1. **Folding.** No design residues are specified, corresponding to structure prediction.
2. **Binder Design.** One protein chain in the target structure is chosen to be designed. This corresponds to binder design against whatever other biomolecules are in the target structure, which could be a small molecule, DNA, RNA, or another protein. Sometimes, only the interface of the protein chain with the rest of the structure is designed, corresponding to binder design with a supplied scaffold.
3. **Motif Scaffolding.** Either a crop of the target structure is chosen to be designed, or everything but the crop is designed. This corresponds to completing a scaffold in the first case, and scaffolding a motif in the second case.
4. **Unconditional Design.** Everything is chosen to be designed, corresponding to unconditional protein generation.

Each task is sampled with some probability during training as long as the target structure is appropriate for the task. For example, the binder design tasks are not sampled for structures that contain a single protein monomer.

In addition to the tasks, we also sample different conditioning to supply the model:

1. **Structure Conditioning.** We randomly choose portions of the target structure to supply as input to the model based on crops. These are also randomly grouped together.
2. **Binding Site.** We randomly annotate residues as being part of a binding site or not, based on proximity to any portions of the training structure selected to be designed.
3. **Secondary Structure.** We annotate random residues by their secondary structure.

See Appendix B.3 for full details on the various schemes we use to construct the tasks and sample the different conditioning inputs to give the model.

### 3.4 Generation

To sample from the model, the inputs are first passed through the trunk to obtain the token and pair representations z that condition the diffusion module. The diffusion module then generates a structure x using the stochastic sampler from [Karras et al., 2022]. This sampler starts from random noise and alternates between adding noise and denoising.


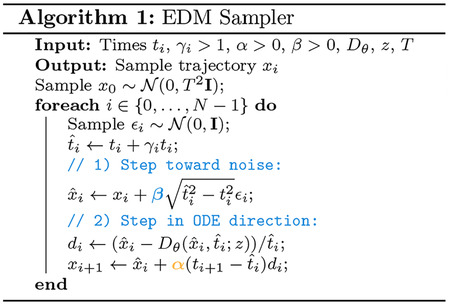


The sampler makes use of the probability flow ODE, which in the case of our forward process is,

(11) $$x^{\prime }\left(t\right)=-\frac{x-\mu_{t}\left(x\right)}{t}dt,$$

for t>0, where $\mu_{t}$ is the posterior mean in Eqn. 2 that is approximated by the denoiser $D_{\theta }$. Note that this ODE runs forward (towards noise). Let F(x,t,s), be the associated flow map, i.e. if x(t) is a solution to the above ODE, then

(12) F(x(t),t,s)=x(s).

The probability flow ODE satisfies that $F\left(X_{t},t,s\right)$ has the same marginal distribution as $X_{s}$. In particular, $F\left(X_{t},t,0\right)$ gives samples from the data distribution for any t>0. Rather than simulate the ODE the whole way, the sampler in [Karras et al., 2022] interleaves noising steps to add stochasticity based on the observation that $X_{t}=X_{s}+\epsilon ,\epsilon \sim 𝒩\left(0,\sqrt{t^{2}-s^{2}}\right)$ for t>s. Hence, alternating between adding noise ϵ and applying the flow map F always gives samples with the correct marginals. The algorithm is given in Alg. 1 which additionally makes use of a step scale α and noise scale β. To sample more high-designability but lower diversity binder structures, we can increase the step scale or decrease the noise scale (and the opposite to obtain more diverse proteins). When drawing many samples, we vary the step scales and noise scales for each generated protein.

#### Dilated schedule.

The time schedule $t_{i}$ used in AlphaFold3 and Boltz is,

(13) $$t_{i}=\sigma_{\text{data}}\cdot {\left(s_{\text{max}}^{1/p}+\tau_{i}\cdot \left(s_{\text{min}}^{1/p}-s_{\text{max}}^{1/p}\right)\right)}^{p},$$

where $\sigma_{\text{data}}=16,s_{\text{min}}=4\cdot 10^{-4},s_{\text{max}}=160,p=7$, and ${\left\{\tau_{i}\right\}}_{i=1}^{N}\in [0, 1]$ is a sequence of steps. By default, $\tau_{i}=i/N$.

Because of our geometric encoding, we found that the amino acid types of designed residues were determined within a short window, approximately at $\tau_{i}\in [0.6,0.8]$. In order to spend more function evaluations when generating the residue types, we therefore use a *dilated* schedule where the interval [0.6, 0.8] is stretched out. Concretely, to dilate an interval $\left[\tau_{s},\tau_{e}\right]$ by $1<\lambda <1/\left(\tau_{e}-\tau_{s}\right)$, we map step τ∈[0, 1] according to

(14) $$\phi (\tau )=\left\{\begin{array}{ll} \tau /r, & \text{if}\tau <l\\ (\tau -u)/r+u & \text{if}\tau >u\\ (\tau -l)/\lambda +l & \text{otherwise} \end{array},\right.$$

where $r=\left(1-\lambda \cdot \left(\tau_{e}-\tau_{s}\right)\right)/\left(1-\left(\tau_{e}-\tau_{s}\right)\right),l=r\cdot \tau_{s},u=l+\lambda \left(\tau_{e}-\tau_{s}\right)$. In practice, we choose $\lambda =8/3,\tau_{s}=0.6$, and $\tau_{e}=0.8$, with N=300 total function evaluations which we found to work well in experiments.

### 3.5 BoltzGen Pipeline

On top of the generative model, we run a computational pipeline to filter from the potentially thousands of designs sampled by the model down to the most promising candidates. The pipeline consists of the following stages.

1. **BoltzGen Diffusion (GPU).** Given a design specification, we generate a large number of designed binders with BoltzGen.
2. **Inverse Folding (GPU).** We optionally inverse fold the designed binders. This step tends to create sequences that are more likely to be predicted to refold into the designed structure. It is also likely to improve solubility (the inverse folding model was only trained on soluble proteins). We use BoltzIF (detailed below), which is similar to SolubleMPNN [Goverde et al., 2024].
3. **Folding (GPU).** We predict the structure of the design in complex with the target using Boltz-2, which is provided the template of the target structure (produced in step 1) and no MSAs. We compute the RMSD between the predicted structure and the structure produced by step 1 for later filtering (if the refolded structure is similar to the designed structure, the design is more likely to be a binder). This also produces confidence metrics (pTMs, pAEs) used in filtering. In scenarios where we design globular proteins, we perform an additional refolding step where we only predict the structure of the designed binder (in absence of the target), and compute the same RMSD metrics. As detailed in D, we found this necessary to assess whether the designed protein’s structure can be achieved in absence of the target, indicating that the design can express well and fold on its own.
4. **Affinity Prediction (GPU).** When designing proteins that bind small-molecules, we predict their affinity using Boltz-2’s affinity module.
5. **Analyze (CPU).** We compute physics-based metrics that provide information about the binder-target interaction strength and developability properties of the design. Here we describe those that are used in the filtering step by default. A complete list is in Appendix B.4. We count the hydrogen bonds and salt bridges between design and target and assess the buried surface area (how much contact is there and how much shape complementarity) between the design and target. Additionally, we compute a sequence based solubility metric and the area of the largest hydrophobic patch on the surface of the designed protein (large hydrophobic patches can cause protein aggregation and difficulty during purification or expression).
6. **Filter (CPU).** Based on the metrics computed in the previous stage, we produce a ranking of the designs (detailed below). This ranking is used in a quality-diversity algorithm (detailed below), which returns a final set of filtered (to a desired budget k), ranked, and diversity optimized candidates. This step takes around 20 seconds, independent of the number of designs.

#### Inverse Folding Model.

Our inverse folding model is largely similar to SolubleMPNN [Goverde et al., 2024]. The main differences are that (1) we use 6 encoder layers instead of the 3 of SolubleMPNN, (2) we exclude fibril proteins from the training set next to excluding transmembrane proteins and (3) we train on structures cropped to 1024 amino acids rather than excluding larger instances. We verify that our inverse folding model performs similar to ProteinMPNN and SolubleMPNN in Section C.3.

#### Ranking.

To rank designs, we first compute two groups of metrics that we expect to correlate with experimental success: Boltz-2 confidence scores [Passaro et al., 2025] and interaction-type scores such as the number of hydrogen bonds. These are aggregated into a single quality score q by taking the worst rank across all metrics (Algorithm 2). Metrics are given varying importance by weighting their respective ranks, where the weights are calibrated on a benchmark of validated binders (see Appendix C.1). This prioritizes designs with the best worst-case performance across all metrics.


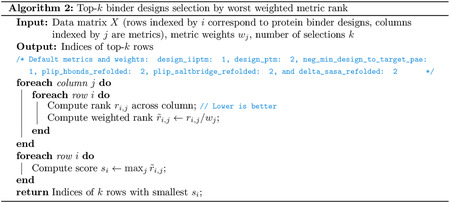


#### Quality-Diversity Selection.

In order to select a *diverse* set of high-ranking designs, we use a greedy selection algorithm described in Alg. 3. Each design x produced by the model is ranked according to the aggregated score q(x) introduced above. For a given set of candidates S, we also measure the similarity of design x to all designs in the set S based on a mixture of the TM-score and sequence similarity,

(15) $$\text{Diversity}(x,A)=1-\left(w_{\text{struct}}\cdot \text{StructSim}(x,A)+w_{\text{seq}}\cdot \text{SeqSim}(x,A)\right),$$

where

(16) $$\text{StructSim}(x,A)=\underset{a\in A}{\text{max}}\;\text{TM-score}(x,a),$$

and

(17) $$\text{SeqSim}(x,A)=\underset{a\in A}{\text{max}}\frac{\text{alignment}(x,a)}{\text{max}(\text{len}(x),\text{len}(a))}.$$

The algorithm then proceeds by greedily adding designs that maximize a combination of quality and diversity with respect to the current set of candidates.


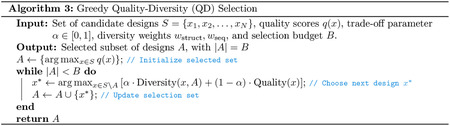


## 4 Detailed Wetlab Results

### 4.1 Designing Nanobodies and Proteins against 9 Novel Targets

Experiments carried out by Adaptyv Bio.

The target choice, design process, and results are described in Section 2.2. Here we provide Tables 3 and 2 to list all attained affinity measurements and describe the targets’ therapeutic and translational relevance in Section 4.1.1. All sensograms, collected datapoints for computing affinities, and associated experimental information is available at: https://huggingface.co/datasets/boltzgen/adaptyv_data1/resolve/main/adaptyv_data.zip

#### 4.1.1 Therapeutic and Translational Relevance of 9 Novel Targets

While largely chosen for benchmarking generalization, many of our 9 “hard” targets also play important roles in disease pathways, therapeutic mechanisms, or emerging biotechnological and synthetic biology applications. Designing binders against them could therefore enable new ways to modulate, inhibit, stabilize, or study these proteins in both therapeutic and translational contexts. We should note that, for intracellular targets, we assume that nanobody-based intrabodies, genetically encoded and expressed within cells, offer a promising modality for studying and modulating protein function *in vivo*, including within organelles such as peroxisomes [Wagner and Rothbauer, 2020].

Some of the targets are directly implicated in chronic inflammation and cancer. For instance, orosomucoid-2 (**ORM2**) is an acute-phase glycoprotein that promotes cytokine production in rheumatoid arthritis synovial tissue [Kim et al., 2024]. Binders that block ORM2-mediated immune signaling could serve as potential anti-inflammatory agents or probes to dissect its role in autoimmune disease. Similarly, **MZB1**, an ER-resident co-chaperone involved in antibody secretion and calcium regulation, is overexpressed in multiple myeloma and chronic lymphocytic leukemia [Chanukuppa et al., 2020]. A binder that perturbs MZB1 folding function could interfere with the secretory machinery of transformed plasma cells and thus represent a novel intervention for B-cell malignancies.

Several of our targets are enzymes whose dysfunction causes inherited or acquired metabolic disorders. Phytanoyl-CoA hydroxylase (**PHYH**) is a peroxisomal enzyme required for phytanic acid breakdown. While mutations in PHYH cause Adult Refsum disease, recent studies have also implicated PHYH in cancer metabolism and other contexts of metabolic dysregulation [Zhengqi et al., 2021]. A highly specific binder could serve as a research tool to selectively modulate or visualize PHYH activity and to study the cellular consequences of impaired alpha-oxidation. Another example is riboflavin kinase (**RFK**), which catalyzes the rate-limiting step in the biosynthesis of FMN and FAD, cofactors essential in redox metabolism. Species-specific differences between human and microbial RFK enzymes support the development of microbial RFK-targeting binders as potential antimicrobial agents [Anoz-Carbonell et al., 2020]. Other enzymes in our panel link directly to cancer metabolism. Phosphomevalonate kinase (**PMVK**) is a critical enzyme in the mevalonate pathway, and recent work shows it drives tumor progression via metabolite-dependent activation of Wnt/β-catenin signaling [Chen et al., 2023]. Designed binders could serve to inhibit this signaling axis or act as pathway-specific probes. **IDI2** is also part of isoprenoid biosynthesis and widely used in engineered microbes to boost terpenoid and carotenoid yields [Farhi et al., 2011]. Tunable binders for IDI2 could thus be applied in enzyme control or stabilization in synthetic biology workflows.

Some of our targets are involved in signaling and regulatory processes relevant to inflammation, neurobiology, and RNA metabolism. **HNMT**, which methylates histamine, plays a central role in histamine clearance and is genetically associated with asthma, allergy, and various neurological traits [Szczepankiewicz et al., 2010, Yoshikawa et al., 2019]. Increasing brain histamine levels through novel HNMT inhibitors could offer therapeutic potential for certain neuropsychiatric conditions [Yoshikawa et al., 2019]. In contrast to HNMT’s role in small-molecule metabolism, **METTL16** is an m^6^A RNA methyltransferase that regulates MAT2A mRNA splicing and S-adenosylmethionine homeostasis. Its functional roles span both RNA processing and metabolic control. Recent studies highlight its context-dependent behavior in cancer: METTL16 can promote tumor progression in colorectal and gastric cancer [Wei et al., 2023, Wang et al., 2021], while acting as a tumor suppressor in bladder and papillary thyroid cancers, where its downregulation correlates with more aggressive disease [Yu et al., 2024, Li et al., 2024]. This functional duality underscores the potential of selective binders as orthogonal tools to dissect METTL16 pathway wiring and regulatory logic across tissue types [Qi et al., 2023].

Finally, two of our targets highlight distinct modes of translational value. **GM2A**, a lysosomal activator protein required for ganglioside GM2 degradation, is deficient or functionally compromised in the AB variant of GM2 gangliosidosis, which leads to neurodegeneration [Renaud and Brodsky, 2015, Conzelmann and Sandhoff, 1978]. Stabilizing or activating binders could therefore serve therapeutic or diagnostic functions in this rare disease, while inhibitory binders may have utility for other neurodegenerative diseases such as Alzheimer’s Disease, where GM2A activity is elevated [Hsieh et al., 2022]. Alpha-1-microglobulin/bikunin precursor (**AMBP**) is a complex plasma glycoprotein that is processed into two distinct, functional proteins: alpha-1-microglobulin (A1M) and bikunin. A1M is a radical-scavenging and heme-binding protein that protects against oxidative stress and tissue injury, while bikunin is a Kunitz-type protease inhibitor involved in extracellular matrix stabilization and inflammation control. Recent work in a preeclampsia mouse model showed that recombinant A1M can alleviate placental and renal oxidative damage, reduce hypertension, and preserve organ function, underscoring its therapeutic potential in pregnancy-related disorders and other oxidative pathologies [Erlandsson et al., 2019]. Binders that selectively enhance or inhibit cleavage, secretion, or the extracellular interactions of AMBP products could be developed as tools to modulate protease activity, track oxidative damage *in vivo*, or regulate post-translational processing of these multifunctional proteins.

In sum, the 9 hard targets selected here are not merely difficult in structural terms — they are underexplored yet promising proteins across therapeutic, diagnostic, and synthetic biology domains. The ability to design functional binders against them suggests new experimental and translational tools that could complement or extend small-molecule and protein binder–based strategies.

### 4.2 Designing Proteins to Bind Bioactive Peptides with Diverse Structures

Experiments by A. Katherine Hatstat, Angelika Arada, Nam Hyeong Kim, Ethel Tackie-Yarboi, Dylan Boselli, Lee Schnaider, and William F. DeGrado.

We sought to test the capacity of BoltzGen to generate binders of biologically active peptides with diverse secondary structures and antimicrobial and/or cytotoxic activity. We targeted three peptides with diverse secondary structures: protegrin (disulfide stapled beta-hairpin) [Gidalevitz et al., 2003, Steinberg et al., 1997], melittin (amphipathic helix in the presence of membranes) [DeGrado et al., 1982, Dempsey, 1990, Guha et al., 2021, Hristova et al., 2001, Vogel and Jähnig, 1986], and indolicidin (polyproline II or amphipathic conformation in the presence of bilayers) [Falla et al., 1996, Ladokhin et al., 1997, 1999]. In this campaign, we define a successful design as one that expresses at high levels in Escherichia coli, is monomeric with the desired secondary structure, and binds its desired target with at least single-digit μM affinity. Because all the binding targets are antimicrobial peptides (AMPs), we also screened binders for their ability to neutralize antimicrobial activity against Bacillus subtilis and, where applicable, their ability to inhibit peptide-induced hemolysis. The former also serves as a measure of the designs’ proteolytic stability as B. subtilis secretes numerous proteases.

From the top ranked designs produced by BoltzGen, we selected six per peptide through manual inspection, prioritizing those with consistent burial, well-oriented hydrogen bonds, and tightly packed interfaces for experimental characterization.

#### Target Peptide Melittin.

**Figure 13:**
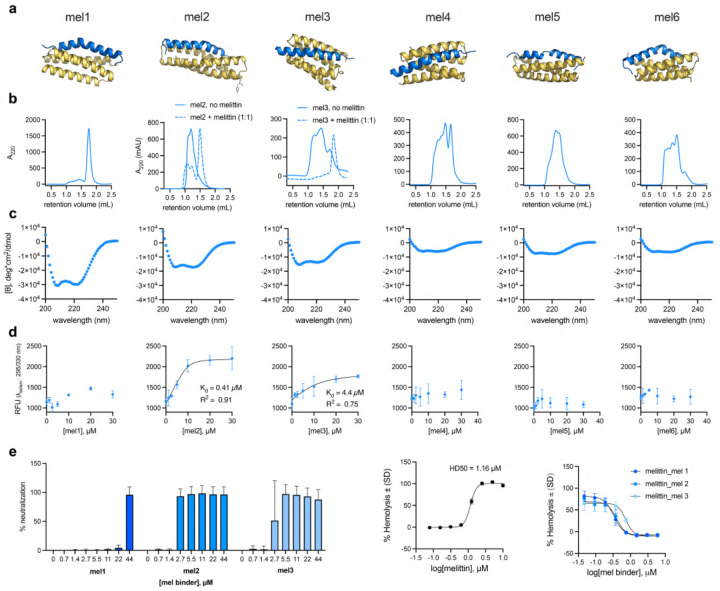
Experimental characterization of melittin binder designs. (**a**) Structure prediction models of all selected melittin binders are predicted to adopt a helical conformation and to bind melittin in its amphipathic helix state. (**b**) Analytical size exclusion chromatography traces of Mel1–Mel6 show varied oligomeric states. (**c**) Circular dichroism shows that Mel1–3 have helical character, while Mel4–6 are only partially folded. (**d**) Mel2 and Mel3 show detectable melittin binding as measured by changes in intrinsic fluorescence of melittin’s tryptophan residue. Melittin concentration is held constant at 10μM while binders, which lack tryptophan residues, are varied from 0-40μM. (e) Mel1–3 neutralize melittin’s antimicrobial activity against *B. subtilis*. (**f**) Mel1–3 also neutralize melittin’s hemolytic activity.

We first evaluated melittin binders for their ability to bind melittin and neutralize its antimicrobial and hemolytic activity. All six selected melittin binder designs (termed mel1–mel6) expressed, and mel1–3 displayed the expected helical structure (Figure 13a,b; $[\Theta {]}_{222,\text{mel1}}=-29, 900\text{deg}*{\;\text{cm}}^{2}/\text{dmol};[\Theta {]}_{222,\text{mel}2}=-17, 200\text{deg}*{\;\text{cm}}^{2}/\text{dmol};[\Theta {]}_{222,\text{mel}3}=-13, 600\text{deg}*{\;\text{cm}}^{2}/\text{dmol}$). Size exclusion chromatography (SEC) showed that mel1 forms a monodisperse species consistent with a monomer, while mel2 and mel3 tended to aggregate (Figure 13c). However, pre-incubation of mel2 and mel3 with melittin before SEC analysis shows that melittin binding acts as a switch, driving mel2 and mel3 from an aggregated state to monodisperse, monomeric species. Mel4–6 show poor folding and form aggregates in SEC (Figure 13b,c).

Mel1–3 were moved forward for assessment of melittin binding through a combination of *in vitro* binding assays and neutralization assays (both antimicrobial and hemolysis assays, as melittin has both antimicrobial activity and cytotoxicity) (Figure 13e,f). All three binders neutralize antimicrobial activity (Figure 13e) and hemolysis (Figure 13f). A single molar equivalent of Mel1 and Mel2 reversed the cytotoxic effect of melittin near its HD50 (1.2μM) for hemolysis of erythrocytes. Melittin contains a single tryptophan residue that is predicted to be near or fully buried in the peptide:binder interface in the designed complexes. Thus, we complemented neutralization assays by monitoring changes in intrinsic tryptophan fluorescence *in vitro* to quantify design binding affinity. Mel2 and mel3 have low to sub-μM affinity for melittin (0.41 and $4.4\;\mu \text{M}\;K_{d}$ for mel2 and mel3, respectively), while mel1 showed limited spectral shift at the excitation and emission wavelengths monitored.

#### Target Peptide Indolicidin.

**Figure 14:**
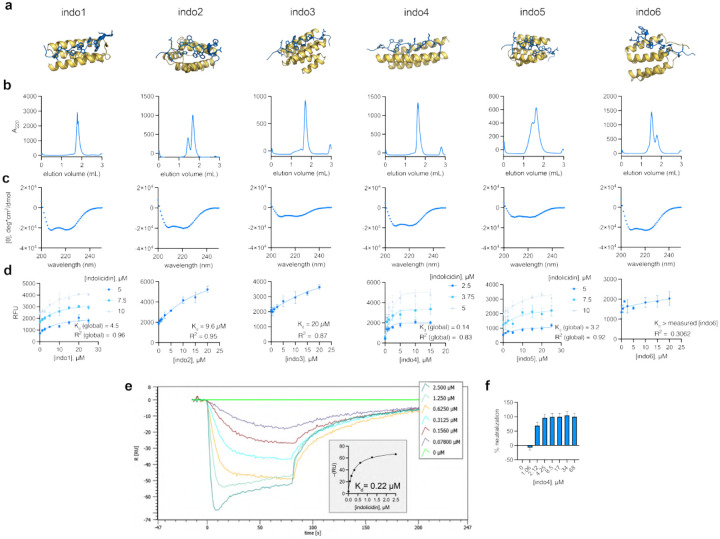
Experimental characterization of indolicidin binder designs. (**a**) Structure prediction models of all selected indolicidin binders are predicted to adopt a helical conformation. (**b**) Analytical size exclusion chromatography traces of Indo1–Indo6 show varied oligomeric states. (**c**) Circular dichroism shows that Indo1–6 all have helical character. (**d**) All Indo binders show detectable indolicidin binding as measured by changes in intrinsic fluorescence of indolicidin’s tryptophan residues. Indo1, Indo4, and Indo5 binding was measured with multiple indolicidin concentrations, and $K_{d}$ was determined via a global fit. (**e**) Only Indo4 neutralizes indolicidin antimicrobial activity against *B. subtilis*. (**f**) For Indo4, binding was validated by surface plasmon resonance, in which Indo4 was immobilized and exposed to varied [indolicidin]. Affinity was determined as a function of response units at the pre-injection stop points of each [indolicidin] (inset).

All selected indolicidin binder designs were predicted to be helical, comprised of 3–6 helices with a peptide binding cleft on the surface of the helical binder (Figure 14a). Three of the six (indo1, indo3, and indo4) designs formed single, homogenous species by analytical size exclusion chromatography, while indo2, indo5, and indo6 tended to aggregate (Figure 14b). All six binder designs had helical character as measured by circular dichroism (Figure 14c; $[\Theta {]}_{222,\text{indo1}}=-21, 700\text{deg}*{\;\text{cm}}^{2}/\text{dmol}$; $[\Theta {]}_{222,\text{indo2}}=-19, 800\text{deg}*{\;\text{cm}}^{2}/\text{dmol}$; $[\Theta {]}_{222,\text{indo3}}=-8, 700\text{deg}*{\;\text{cm}}^{2}/\text{dmol}$; $[\Theta {]}_{222,\text{indo}4}=-17, 700\text{deg}*{\;\text{cm}}^{2}/\text{dmol}$; $[\Theta {]}_{222,\text{indo}5}=-9, 600\text{deg}*{\;\text{cm}}^{2}/\text{dmol}$; $[\Theta {]}_{222,\text{indo6}}=-22, 900\text{deg}*{\;\text{cm}}^{2}/\text{dmol}$). All indolicidin binder designs showed indolicdin binding as monitored by changes in indolicidin tryptophan fluorescence (Figure 14d; $K_{d,\text{indo4}}<K_{d,\text{indo1}}<K_{d,\text{indo5}}<K_{d,\text{indo2}}<K_{d,\text{indo3}}<K_{d,\text{indo6}}$), with indo 1, 3, and 5 exhibiting affinities <5μM and indo4 exhibiting sub-μM affinity. (Figure 14d). The indo4:indolicidin interaction was further analyzed by surface plasmon resonance, which confirmed that the complex binds with nanomolar affinity (Figure 14e). Despite all six designs having detectable indolicidin binding, only indo4 showed robust neutralization of indolicidin antimicrobial activity (Figure 14f). This may be due to low proteolytic stability of the designs in the presence of B. subtilis, which secretes an array of proteases [van Wely et al., 2001].

#### Target Peptide Protegrin.

**Figure 15:**
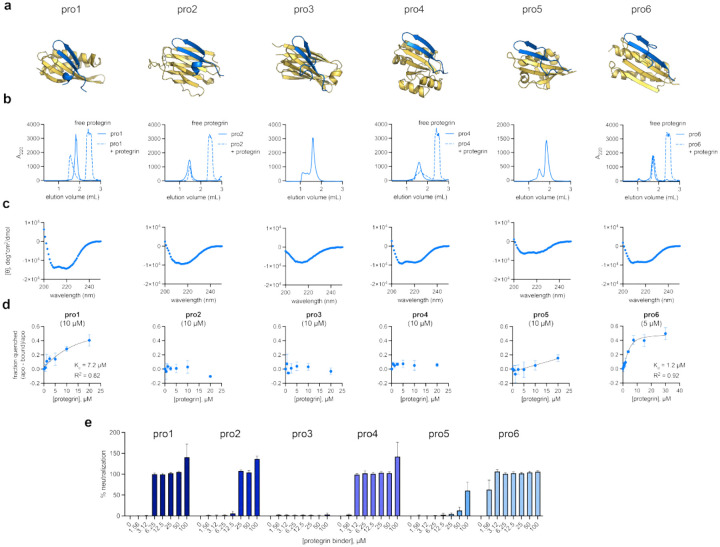
Experimental characterization of protegrin binder designs. (**a**) Structure prediction models of all selected protegrin binders are predicted to adopt a helical conformation. (**b**) Analytical size exclusion chromatography traces of Pro1–Pro6 show varied oligomeric states. (**c**) Circular dichroism shows that Pro1, 4, 5, and 6 have partial helical character, while Pro2 and Pro3 have the expected β-structure in their apo form. (**d**) Pro1, 5, and 6 show detectable indolicidin binding as measured by changes in intrinsic fluorescence of the binders’ tryptophan residues. (**e**) Pro1, 2, 4, and 6 fully neutralize protegrin antimicrobial activity against *B. subtilis* at the concentrations tested.

Finally, we assessed the ability of BoltzGen to generate binders to protegrin, a disulfide-stapled, beta-hairpin antimicrobial peptide. Unlike the previous targets, the generated designs for protegrin binders showed mixed alpha/beta or all beta topologies (Figure 15 a). By SEC, pro1 and pro6 formed single monodisperse species consistent with a monomeric state, while pro2–5 eluted at volumes consistent with higher-order oligomers or mixed oligomeric states (Figure 15 b). All six designs showed the expected secondary structure by circular dichroism (beta or mixed alpha/beta) (Figure 15 c). Two of the six designs, pro1 and pro6, showed detectable binding to protegrin via changes in binder tryptophan fluorescence, with affinities of 7.2 and 1.2μM, respectively (Figure 15 d). Both pro1 and pro6 neutralized protegrin, with pro6 being a more potent neutralizer. This is consistent with the relative affinities of the two designs as measured by tryptophan fluorescence changes. While pro2, pro4, and pro5 showed little spectral shift in tryptophan fluorescence assays, they all neutralized protegrin’s activity against B. subtilis. The trp residues in these designs are not predicted to be fully buried in the bound complex; thus, neutralization assays indicate a binding event is occurring but it may not be detectable by the *in vitro* binding assay used herein (Figure 15 e).

### 4.3 Designing Peptides to Bind the Disordered Region of NPM1.

Experiments by Yaotian Zhang, and Denes Hnisz.

#### Designs.

Using BoltzGen, we generate 20,000 designs of size 40–80 to bind NPM1’s disordered region and test 5 designs experimentally. We make use of BoltzGen’s binding site conditioning and specify that the design should interact with the disordered region and *not* its structured beta-sheet region via our model’s “not-binding” feature. Additionally, we provide the structure of the ordered region and leave the disordered region flexible. Thus tasking BoltzGen to model how the disordered region will fold and become structured in presence of the peptide while designing that peptide.

#### Detailed Results.

**Figure 16:**
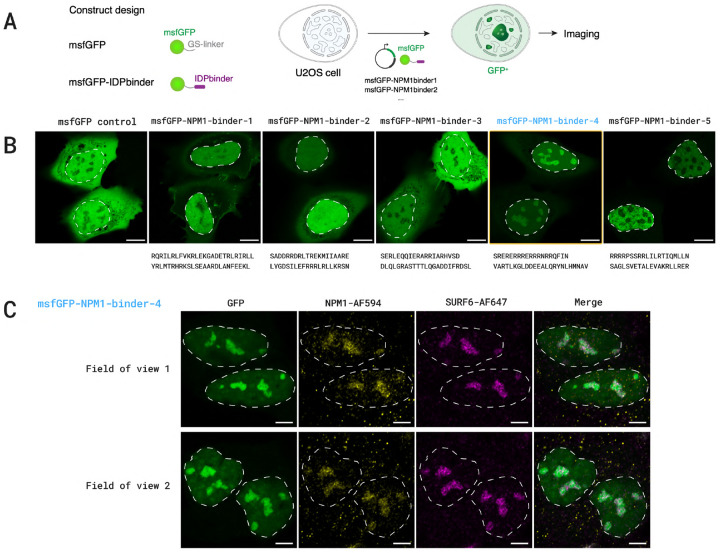
A. Schematic model of the msfGFP-tagged binder design and cellular assay to visualize subcellular localization of the NPM1-binders. B. Live cell fluorescence microscopy images of U2OS cells expressing ectopic msfGFP-NPM1 binders. The cell nucleus is highlighted with a dashed white line contour. Scale bar: 10μm. The experiment was repeated twice independently with similar results. C. Fixed-cell immunofluorescence of U2OS cells expressing exogenous msfGFP-NPM1-binder-4. Endogenous NPM1 and SURF6 are stained with antibodies. The cell nucleus is highlighted with a dashed white line contour. Scale bar: μμm. The experiment was repeated twice independently with similar results.

We assessed the target engagement of the NPM1-binders in live cells. Five selected NPM1-binders were genetically fused to GFP, and the GFP-tagged binders were transiently expressed in human osteosarcoma (U2OS) cells. The subcellular localization of the binders was visualized with GFP fluorescence (Figure 16 A). Among the five tested binders, one binder (NPM1-binder-4) showed localization consistent with enrichment in nucleoli where the endogenous NPM1 protein is known to localize (Figure 16 B). Immunofluorescence staining for NPM1 indeed confirmed the colocalization of the GFP-tagged NPM-binder-4 with endogenous NPM1 (Figure 16 C), thus providing evidence of a de-novo designed protein binding disordered proteins *in live* cells. As an additional control for nucleolar localization, another well-known protein that also localizes in nucleoli, SURF6, was also visualized (Figure 16 C).

### 4.4 Designing Peptides to Bind a Specific Site of RagC GTPase

Experiments by Shamayeeta Ray, Jonathan T. Goldstein, and David M. Sabatini.

#### Target and Designs.

All information is already present in the summary Section 2.5.

#### Results.

We test 29 designs in an SPR assay and find 7 binders with affinities ranging from 3.5μM to 893μM (Table 4). Additionally, for 4 binders, we randomly permute their residues and re-test binding. Three lose their affinity and 1 shows 10× weaker binding. Furthermore, the permuted version of peptide 23 showed poor binding at lower concentration and displayed uninterpretable sensograms at concentrations greater 25μM, indicating non-specific binding.

### 4.5 Designing Disulfide Bonded Cyclic Peptides to Bind a Specific Site of RagA:RagC

Experiments by Shamayeeta Ray, Jonathan T. Goldstein, and David M. Sabatini.

#### Target and Designs.

All information is already present in the summary Section 2.6.

#### Results.

We test 24 peptides and find 14 of them to show specific binding by SPR. For 8 of those we resolved the affinities obtaining values ranging from 80μM to 1100μM (Table 5).

### 4.6 Designing Nanobodies that Bind Penguinpox and Hemagglutinin

Experiments by Jacob A. Hambalek, Anshika Gupta, Diego Taquiri Diaz, and Chang C. Liu.

#### Targets.

All information is already present in the summary Section 2.7.

#### Designs and Results.

We generate 60 000 nanobodies against each target in the fashion described in Section 2.2 and select 7 per target for experimental characterization. Of the 7 designed nanobodies that we tested for each antigen through yeast surface display at a range of antigen concentrations (7nM-2μM), we observed a weak binding signal at the highest antigen concentration (1.6-2μM) for some designs. Specifically, 1 of 7 designs against cGAMP PDE showed a binding signal at 1.6μM of the antigen, and 7 of 7 designs against FhaB—where all 7 designs were against the same epitope—showed a binding signal at 2μM of the antigen. Because 2μM was the highest antigen concentration used for labeling and was the minimal concentration tested that resulted in any binding signal, we cannot calculate an EC_50_ from these studies. However, we can conclude that the binders exhibiting weak binding signals are at best 2μM affinity binders.

**Figure 17:**
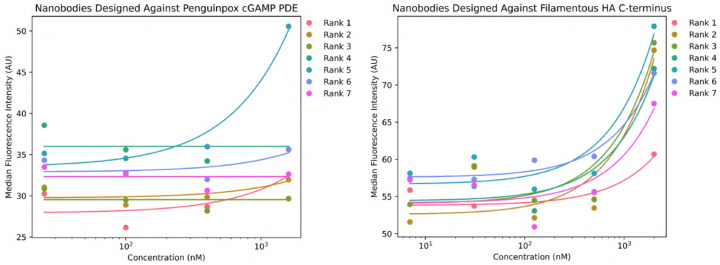
YSD of nanobodies for feasibility assessment of design optimization. Median fluorescence intensities above background for cells displaying nanobodies targeting (**left**) cGAMP PDE and (**right**) FhaB. The highest concentration of antigen in both cases was the only test case that demonstrated any binding signal for any nanobody design.

The plots in Figure 17 represent the median Alexa Fluor 647 fluorescence intensity of nanobody-displaying cells (HA tag label positive) minus the median Alexa Fluor 647 fluorescence intensity of the non-nanobody-displaying cells (HA tag label negative) at different concentrations of antigen. The latter acts as an internal control and is subtracted to remove the signal from nanobody-independent background binding of cells to antigen. Higher fluorescence intensity indicates more binding. The background-subtracted median fluorescence intensities across concentrations for each nanobody design were then fit to a Hill function using SciPy curve_fit. For initializing curve fitting, the background value used was the minimum fluorescence intensity observed at 0 antigen concentration and the EC_50_ used was the median antigen concentration tested. The Hill coefficient, n, was explicitly set to 1, as we expect non-cooperative binding of the antigen to the antibody. The fluorescence value, Y, is fit as:

$$Y=B_{\text{min}}+\frac{B_{\text{max}}L}{\tilde{C}+L}$$

where $B_{\text{min}}$ is the best-fit background fluorescence value, $B_{\text{max}}$ is the best-fit maximum binding value, $\tilde{C}$ is the best-fit EC_50_ projection, and L is the concentration of antigen.

### 4.7 Designing Proteins that Bind to Small Molecules

Experiments by A. Katherine Hatstat, Angelika Arada, Nam Hyeong Kim, Ethel Tackie-Yarboi, Dylan Boselli, Lee Schnaider, and William F. DeGrado.

**Figure 18:**
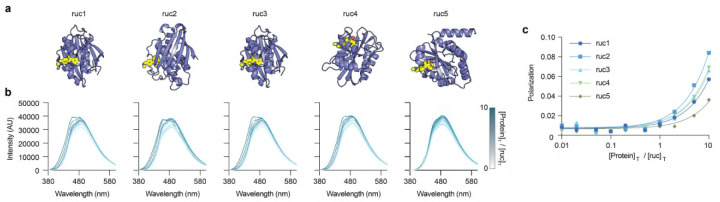
rucaparib Binder Design **a)** Structural model of the designed rucaparib binder (purple) in complex with rucaparib (yellow). **b)** Fluorescence emission spectra of rucaparib (10μM) upon titration with increasing concentrations of the designed protein (0–10 equivalents). The emission spectrum of rucaparib exhibited a blue shift upon binding, indicating changes in its local environment. **c**) Fluorescence polarization assay of rucaparib binding to the designed protein, measured with an excitation wavelength of 405 nm and emission wavelength of 516 nm. Polarization values were plotted against the molar ratio of protein to rucaparib, and the data were fitted to a one-site binding model using nonlinear regression in GraphPad Prism 10 to determine the dissociation constant ($K_{d}$).

We selected rucaparib as a benchmark target, as published reports indicate that high-affinity binders ($K_{d}<5\;\text{nM}$) can be achieved. We also selected a rhodamide derivative related to rucaparib, which we leave undisclosed. Our computational design pipeline generated on the order of 10,000 designs for each target. The initial set was filtered to a highest-confidence subset of 100 designs based on RMSD < 2.5 Å relative to the Boltz-2 refolded models. Within this filtered pool, designs were ranked using a composite metric (interaction score + Boltz score), which effectively integrates predicted structural fidelity with biophysical interaction quality. To identify essential interactions, we fragmented the chemical groups of rucaparib and calculated the number of hydrogen bonds formed with each fragment. We prioritized designs forming hydrogen bonds with the carboxamide chemical groups, as this interaction is considered essential for specific binding. A total of six designs for rucaparib and 4 designs for the rhodamine derivative were selected for experimental validation by binding assay (Figure 18a).

Among the selected rucaparib binder designs (termed ruc1–ruc6), five out of six were expressed in moderate to good yields (15 – 69.5 mg/L). Incubation of each binder with equimolar concentrations of rucaparib led to a marked blue-shift and an increase in the intensity of its fluorescence spectrum, which is characteristic of the rucaparib indole core being bound in a rigid, solvent-inaccessible site (Figure 18b). Fluorescence polarization data showed that ruc1–ruc4 exhibited moderate affinity for rucaparib, with values of 75.9, 43.0, 64.2, and $59.1\;\mu \text{M}{\;K}_{d}$, respectively. Ruc5 showed the weakest binding affinity ($151.5\;\mu \text{M}{\;K}_{d}$) among the tested candidates (Figure 18c). All designs against the rhodamine derivative expressed in moderate to good yields (15 – 69.5 mg/L), and fluorescence polarization data showed binding affinities of 30.9, 69.2, 144.7 and 252.2μM. Affinities of all designs against both targets are summarized in Table 6.

### 4.8 Designing Antimicrobial Peptides that Inhibit the GyrA to GyrA Interaction

Experiments by Andrew Savinov, and Gene-Wei Li

Recent work has demonstrated that peptide fragments of full-length proteins (protein fragments) are generalizable inhibitors of native protein interactions in living cells [Savinov et al., 2022, 2025]. Libraries of tiling protein fragments also reveal specific interfaces prone to such protein fragment-based inhibition [Savinov et al., 2022], identifying good target sites for alternative drug design modalities.

Here, we sought to leverage previous results uncovering potent inhibitory protein fragments of the essential bacterial protein DNA gyrase (subunit A; hereafter, GyrA), where the C-gate closure interaction between two GyrA subunits was identified as a desirable target site [Savinov et al., 2022, 2025]. DNA gyrase is a target of considerable interest for developing novel antibiotics. Existing compounds, such as clinically employed fluoroquinones, function by trapping gyrase in the DNA-cleavage complex [Khan et al., 2018, Bax et al., 2019] and aminocoumarins interfere with the enzyme’s ATPase activity [Khan et al., 2018], but interfering with C-gate closure represents a complementary drug modality. Indeed, a previously reported monoclonal antibody against *Mycobacterium tuberculosis* GyrA appears to target this same site [Manjunatha et al., 2005].

We therefore used the inhibitory peak identified by fragment scanning experiments [Savinov et al., 2022, 2025] and massively parallel predictions of fragment binding modes with FragFold [Savinov et al., 2025] to identify target residues in the GyrA C-gate closure complex, and used BoltzGen to design *de novo* peptide binders targeting this same interface. We note that this was a challenging target for *de novo* binder design owing to the small size of the C-gate closure interface (i.e., only 6 residues within 4 Å in the experimental structure of the full gyrase complex (PDB ID 6RKS), and 11 residues within 4 Å in the FragFold model [Savinov et al., 2025]) and we therefore designed binders with diverse design parameters. We then leveraged an established experimental method to measure the inhibitory effects of these designed binders in living *E. coli* cells, taking advantage of the importance of GyrA for cell growth to determine inhibitory function from massively parallel measurements of growth inhibition [Savinov et al., 2022, 2025]; in this way a large library of BoltzGen designs was tested in parallel (Methods).

1,808 designed GyrA inhibitors were tested alongside 1,788 mutants designed to break the designed binding modes (3 alanine substitutions at the binding interface per design) to determine the specificity of inhibitory activity to binding GyrA as desired. These designs were tested alongside 30-aa fragments tiling across GyrA with a 1 aa step size, matching previously published work [Savinov et al., 2022, 2025], as an internal positive control for GyrA-inhibitory fragments; and also 20-aa fragments tiling (1 aa step size) across enhanced GFP (eGFP), approximately matching the average fragment length of the designs (15 ± 3.4 aa, mean ± s.d.). The eGFP fragments provided an internal negative control in the form of fragments not expected to interact specifically with *E. coli* proteins.

**Figure 19:**
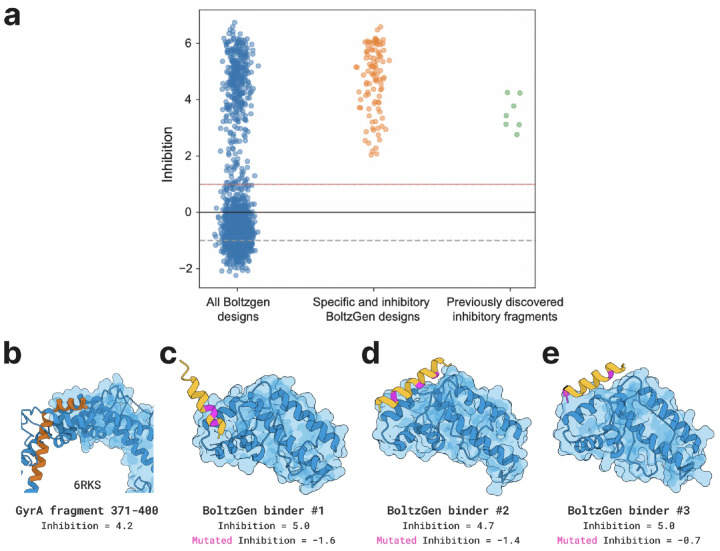
BoltzGen-designed binders are potent DNA Gyrase inhibitors *in vivo*. **(a)** GyrA inhibition from massively parallel in-cell measurements of peptides (Methods) for all designed GyrA binders; designed binders that were inhibitory and specific to the designed binding mode (5.5% of all designs); and inhibitory fragments of GyrA targeting the same site ([Savinov et al., 2022, 2025]). Note that all values for the inhibitory GyrA fragments and 91% of the values for interface-specific designed binders represent lower bounds on inhibitory activity, as these peptides inhibited growth so thoroughly that they completely dropped out of the library when expressed. (**b**) Structure of inhibitory fragment 371–400 of GyrA (orange; [Savinov et al., 2022, 2025]) bound to full-length GyrA (blue) in the context of the full-length GyrA dimer structure (PDB ID 6RKS), with *in vivo* inhibitory effect indicated. **(c) - (e)** Three example BoltzGen-designed binders targeting the same interface as GyrA fragment 371–400, exhibiting high inhibition and specificity *in vivo*. In each case the in-cell inhibitory effects of the designed binder as well as the mutated form with 3 alanine substitutions at the interface (magenta residues) are indicated. In all three cases, inhibitory effects are completely lost upon mutation.

Across all designed GyrA binders tested, 352 (19.5%) were found to substantially inhibit *E. coli* growth, similar to known inhibitory protein fragments of GyrA (Figure 19a–b; Methods). The binders inhibiting growth specifically by binding the GyrA C-gate at the desired interface should generally exhibit loss of activity when residues at the designed interface are mutated (Figure 19c–e), so we calculated the quantity Δ(Inhibition)=Inhibition(design)-Inhibition(mutated design) as a measure of designed GyrA inhibitor specificity. Of all 352 growth-inhibiting designs, 54 designed binders (3.0% of total) were strongly specific for the designed GyrA binding site (Δ(Inhibition)≥2) and 99 designed binders (5.5% of total) were significantly specific under a less stringent requirement (Δ(Inhibition)≥1). Thus, ~3–6% of designed GyrA inhibitors appear to inhibit growth by binding GyrA at the C-gate site as designed, and almost 20% of designs targeting this essential protein inhibited growth more broadly.

The fraction of successfully designed GyrA inhibitors was comparable to the fraction of tiling fragments across GyrA producing inhibitory activity (~9%), reflecting the previously noted widespread inhibitory activity of protein fragments across diverse proteins [Savinov et al., 2022, 2025]. Comparing these results to the effects of expected nonfunctional protein fragments of similar length as represented by the 20 aa eGFP fragment library, we found that 0.45% of eGFP fragments inhibited *E. coli* growth. Therefore, compared to nonspecific control peptides, a ~43-fold larger fraction of designed GyrA binders inhibit cell growth, and ~7–12-fold larger fraction specifically inhibit growth dependent on the designed binding interface.

The inhibitory effects of interface-specific designed binders (Inhibition = 4.7 ± 1.2, mean ± s.d.) were comparable to the inhibitory effects of GyrA fragments from the corresponding inhibitory peak (Inhibition = 3.5 ± 0.6, mean ± s.d.) in internal control measurements in the same experiment (Figure 19a). We note as well that all GyrA-inhibitory protein fragments and 91% of interface-specific designed GyrA binders were sufficiently strong inhibitors of this essential protein that they completely dropped out of the cell population when expressed, meaning these values represent a lower bound on inhibitory activity. The successfully designed DNA gyrase inhibitors represent promising novel antimicrobials targeting this essential bacterial protein.

### 4.9 Designing Nanobodies and Proteins against 5 Benchmark Targets

Experiments carried out by Adaptyv Bio.

The target choice, design process, and results are described in Section 2.10. Here we provide Table 7 to list all attained affinity measurements. The sensograms, from which the affinities were determined, and associated experimental information are available at: https://huggingface.co/datasets/boltzgen/adaptyv_data1/resolve/main/adaptyv_data.zip.

## 5 Computational Results

### 5.1 Structure Prediction

BoltzGen’s development was driven by the hypothesis that designing high-affinity binders requires strong structure prediction and reasoning capabilities. Expressive features that allow for accurate structure prediction are also crucial for design and enable the model to place atoms that tightly interact with the target. Thus, we assess BoltzGen’s structure prediction performance.

**Figure 20:**
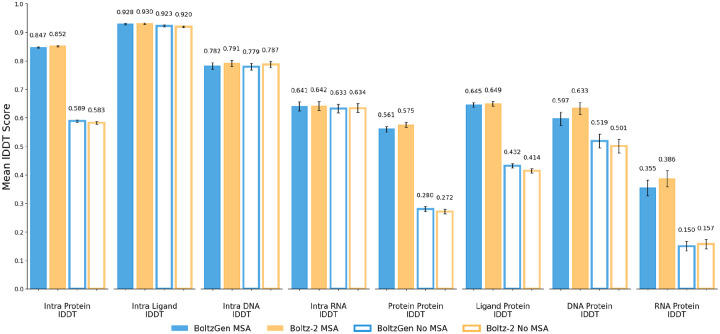
Structure Prediction Evaluation. We reason that structure-based binder design requires strong structure prediction and reasoning capability. BoltzGen can perform design *and* folding. Its folding performance matches Boltz-2. Shown is the best lDDT out of 5 diffusion samples for each complex.

Figure 20 shows that BoltzGen’s folding performance matches Boltz-2 on its test set (minus 187 complexes that did not fit on a 40 GB GPU). This test set is based on clustering sequences with a 40% similarity threshold (details in B.1.2).

### 5.2 Computational Binder Design

In a comparison with RFdiffusion [Watson et al., 2023] and its extension RFdiffusionAA [Krishna et al., 2024] we aim to assess the degree to which the models ignore the target and produce the same structures independent of their conditioning. To evaluate a model’s target dependence, we collect a set of targets and draw one binder design per target and filter that set, only keeping the designs that match the model’s generated structure after refolding with Boltz-2 (based on an RMSD threshold of 2.5 Å). We then assess the diversity of the resulting set as the Vendi score [Friedman and Dieng, 2022] with TM-score as the similarity kernel. Methods that tend to produce the same structures irrespective of the target will obtain a lower diversity.

**Figure 21:**
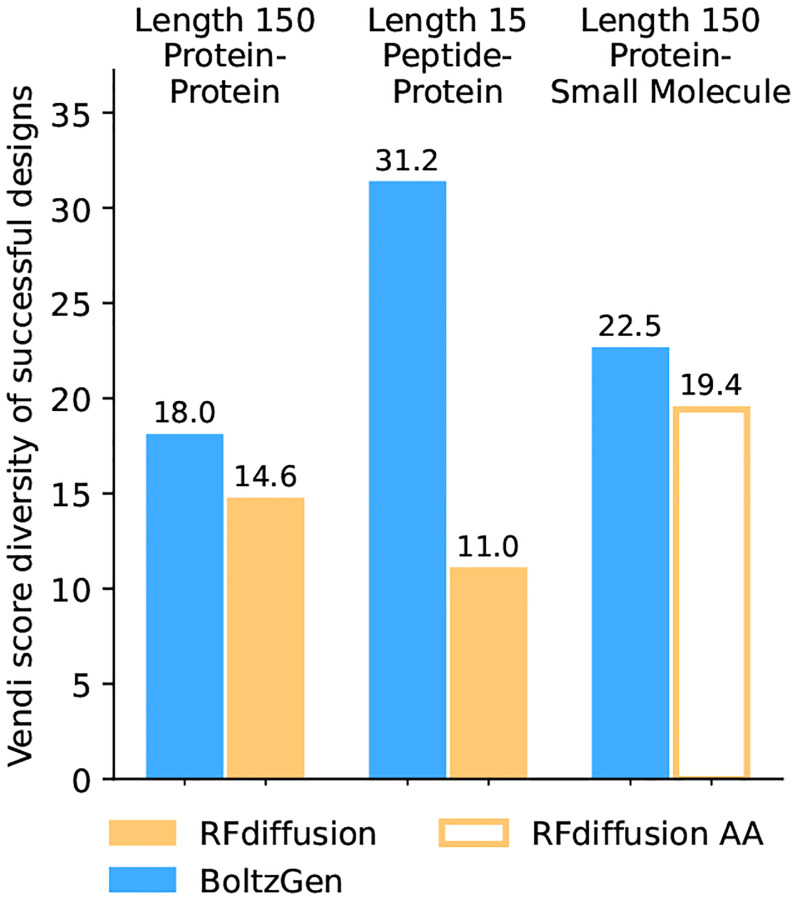
Target conditioning quantification. The diversity of successfully refolded complexes when designing a single binder against 110 targets. This assesses the degree to which the models are conditioned on the target instead of generating designs independent of the target.

We carry this evaluation out for monomeric proteins and small molecules as targets. The monomer set is selected based on sequence similarity clustering and June 2023 as a date cutoffs (details in B.1). The small molecule set is a random sample from all ligands that satisfy standard drug-like property criteria (Lipinski’s rule of five [Lipinski et al., 2001]) and have a Tanimoto similarity of less than 0.35 to any small molecule with the same date cutoff. The comparison in figure 21 shows that BoltzGen “pays more attention” to the target.

## 6 Limitations

For therapeutic development, generating high-affinity binders is only the first step. Whether a design will achieve the intended function depends on a range of additional properties, including selectivity, developability, and the precise characteristics of the target. While predictors for these could be included in BoltzGen’s filtering steps, the *selective* binding problem may be particularly ripe for direct integration into the generative process. Classifier-free guidance techniques could be used for combining the scores of BoltzGen with different targets as conditioning to achieve guiding toward one target while steering away from off-targets. Alternatively, BoltzDesign1 [Cho et al., 2025a] could be used to suggest mutations that prevent binding to off-targets.

More specific to BoltzGen, we note that there is a memorization issue when designing binders of length 73–76. For certain protein targets, BoltzGen’s generation diversity collapses in this length range and it nearly exclusively samples ubiquitin as the binder. For future BoltzGen training runs, ubiquitin should be downsampled (appears more than 900 times in the PDB). More details about this ubiquitin memorization is provided in Appendix C.5.

Lastly, we comment on how there is a tendency in the field to claim that binder design models are “zero-shot” and “plug-and-play” solutions without a chance for failure. We do not make this claim and encourage users to use BoltzGen thoughtfully, carefully inspect the generated structures, and potentially rerun the pipeline multiple times, first at smaller and then larger scales. BoltzGen’s rich design specification language provides a large degree of control that should be experimented with for optimal results.

## 7 Conclusion

BoltzGen is a general-purpose generative model for biomolecular binder design, supporting a broad range of modalities, including proteins, peptides, nanobodies, and related modalities, which can be designed to target virtually any biomolecule, such as proteins, small molecules, and nucleic acids. While the core of BoltzGen is a diffusion generative model, we provide it as part of a broader framework that includes tools for specifying design tasks, generating candidates, and filtering, ranking, and optimizing for diversity. This makes it a practical, end-to-end solution for several binder design problems.

Our strongest results to date are in nanobody design against protein targets, where we obtain nanomolaraffinity binders against two-thirds of the tested novel targets with only 15 or fewer designs. We also demonstrate finding binders when designing miniproteins and peptides against a wide variety of ordered and disordered regions of proteins.

We release the entire BoltzGen package under the MIT license, including model weights, training and inference code, and a user-friendly pipeline for design and evaluation. By providing a complete and accessible solution, we hope BoltzGen serves as a practical tool and a foundation for future work in general-purpose biomolecular design.

## Supplementary Material

1

## Figures and Tables

**Figure 4:**
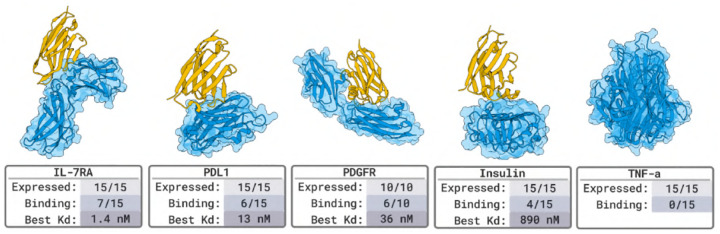
Nanobody binders targeting 5 Benchmark Proteins.

**Figure 5:**
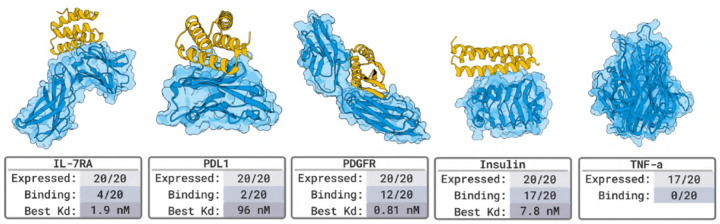
Protein binders targeting 5 Benchmark Proteins.

**Figure 6:**
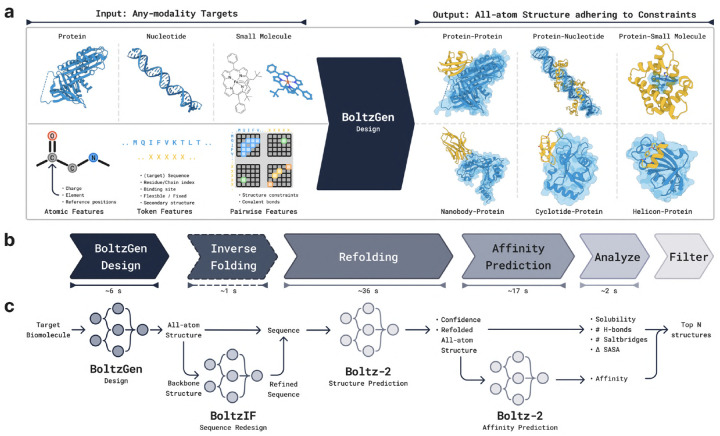
Overview of BoltzGen Pipeline: (**a**) The overall flow from target specification to binder generation. The generative model designs binders for arbitrary targets, including proteins, nucleic acids, and small molecules. It supports various constraints such as covalent bonds, structural motifs, and specific binding sites. The generated designs are passed through a filtering and ranking pipeline to produce a small, diverse set suitable for experimental validation. (**b**) Runtime per stage for a representative case with 200 total residues across the binder and target, illustrating the model’s efficiency. (**c)** A detailed breakdown of each pipeline stage, showing intermediate inputs and outputs.

**Figure 11:**
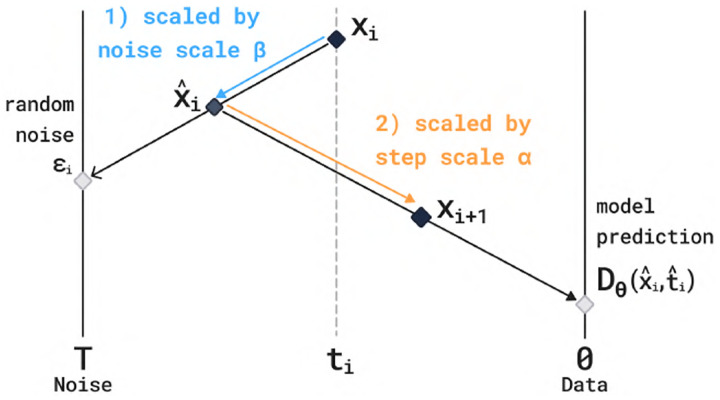
The EDM Sampler steps toward noise and then toward the model prediction in each denoising iteration. The magnitude with which to make these steps can be scaled by β (noise) and α (prediction). Using α≠1,β≠1 no longer samples the training distribution, but is a heuristic scaling to sample more diverse (higher β, lower α) or more designable (lower β, higher α) proteins.

**Figure 12:**
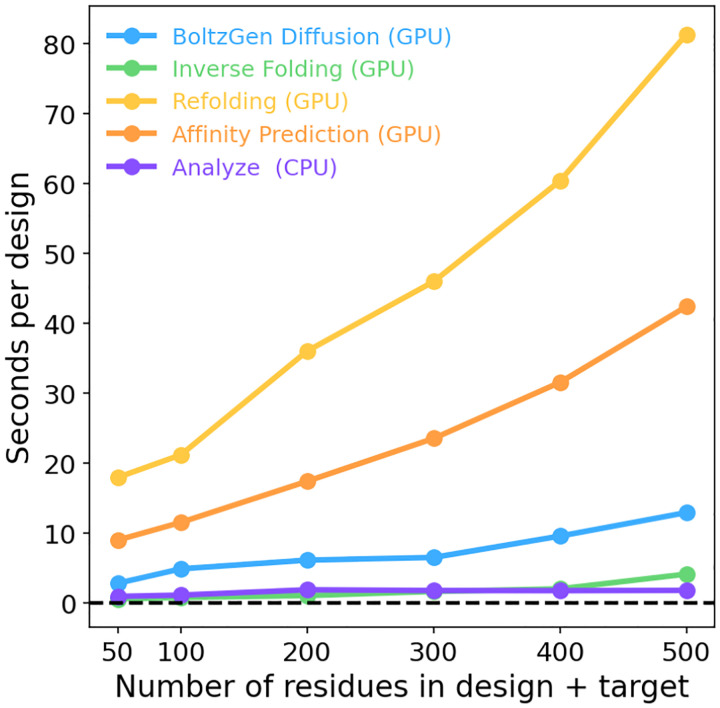
Time consumption of each step in seconds per design for different numbers of residues in the design + target complex (on a single NVIDIA A100 GPU).

**Table 1:** Reported binding affinities $\left(K_{d}\right)$ of therapeutic antibodies and peptides.

Drug	Target	$K_{d}$ (nM)
Caplacizumab	vWF	8.5
Brolucizumab	VEGF-A	0.03
Ozoralizumab	TNFα	0.02
Degarelix	GnRH-R	1.68
Desmopressin	AVPR2	0.76
Tirzepatide	GIPR	0.13

**Table 2:** Affinities for Novel Targets - Nanobody Designs. Entries in blue correspond to the average of 2 replicate measurements, orange corresponds to single measurements. Affinity $K_{D}$ in nM. Expressed designs that do not bind are marked as ◦; lack of expression is marked as ×.

AMBP (3qkg)	GM2A (1g13)	HNMT (1jqd)	IDI2 (2pny)	MZB1 (7aah)	ORM2 (3apu)	PHYH (2a1x)	PMVK (3ch4)	RFK (1nb0)
◦	◦	99	520	120	◦	7.8	6.1	8.8
◦	◦	◦	◦	◦	◦	◦	9.1	18
◦	◦	◦	◦	◦	◦	◦	13	◦
◦	◦	◦	◦	◦	◦	◦	23	◦
◦	◦	◦	◦	◦	◦	◦	66	◦
◦	◦	◦	◦	◦	◦		76	◦
◦	◦	◦	◦	◦	◦		200	◦
◦	◦	◦	◦	◦	◦		440	◦
◦	◦	◦	◦	◦	◦		◦	◦
◦	◦	◦	◦	◦	◦		◦	◦
◦	◦	◦	◦	◦	◦		◦	◦
◦	◦	◦	◦	◦	◦		◦	◦
◦	◦	◦	◦	◦	×		◦	◦
◦	◦	◦	◦	◦	×		◦	◦
◦	×	×	×	◦	×		×	×

**Table 3:** Affinities for Novel Targets - Protein Designs. Entries in blue correspond to the average of 2 replicate measurements, orange corresponds to single measurements. Affinity $K_{D}$ in nM. Expressed designs that do not bind are marked as ◦; lack of expression is marked as ×.

AMBP (3qkg)	GM2A (1g13)	HNMT (1jqd)	IDI2 (2pny)	MZB1 (7aah)	ORM2 (3apu)	PHYH (2a1x)	PMVK (3ch4)	RFK (1nb0)
53	◦	270	26	9.8	◦	22	10	◦
190	◦	◦	66	12	◦	31	12	◦
710	◦	◦	73	14	◦	91	◦	◦
890	◦	◦	230	23	◦	120	◦	◦
*weak*	◦	◦	◦	25	◦	160	◦	◦
◦	◦	◦	◦	25	◦	◦	◦	◦
◦	◦	◦	◦	26	◦	◦	◦	◦
◦	◦	◦	◦	31	◦		◦	◦
◦	◦	◦	◦	31	◦		◦	◦
◦	◦	◦	×	32	◦		◦	◦
◦	◦	◦	×	73	◦		◦	×
◦	×	◦	×	160	×		◦	×
◦	×	◦	×	220	×		◦	×
◦	×	◦	×	310	×		◦	×
◦	×	×	×	◦	×		◦	×

**Table 4:** Binding affinities and response maxima of BoltzGen’s peptide designs. 29 peptides were tested in total. $R_{\text{max}}$ is the saturated signal of a sensogram at maximum binding representing complete occupancy of all ligand sites. RU is Response Units. Scr20 denotes a version of peptide 20 with randomly permuted residues.

Peptide	Sequence	$K_{d}\;(\mu M)$	$R_{\text{max}}$ (RU)
6	ICTLHRK	60	375
13	GVKEDCQALRAQSKALRK	117	249
18	IMTLKRFSKNYGEIERLALY	893	505
19	QLYHIRIARSAQRIFKNGG	268	3041
20	HAMSKNMQRFLRKAKAMVIV	3.5	2083
23	MMSDVRQLRTIVRELRRV	268	285
29	DYSAGRQLLRTLKDKLTTS	373	185
Scr20	KWVHIFRALAMKAMRSQKNM	33.5	451

**Table 5:** Binding affinities and response maxima of BoltzGen’s disulfide-bonded cyclic peptide designs against the RagA:RagC dimer. 24 peptides were tested in total, 14 bind, and for 8 we resolved the affinities. $R_{\text{max}}$ denotes the maximum sensogram signal corresponding to full ligand occupancy. RU: Response Units.

Peptide	Sequence	$K_{d}\;(\mu M)$	$R_{\text{max}}$ (RU)
7	RLRERCRLNPLYCL	308	670
8	RRRERCRLNPLYCG	318	2200
11	SRRERCRLNRLLCLL	447	2000
12	RRRELCKLNPLVCG	1100	1750
14	RRRELCRLDRRACL	297	550
16	KRREACARYRTICLH	164	250
18	IAKRCKADPYRCKLLSR	80	1200
22	GCSKDVQKCKLLK	274	290

**Table 6:** Small Molecule Binders. Experimental characterization of designed binders against rucaparib and an undisclosed rhodamine derivative.

	Length	Expressed	$K_{d}\;(\mu M)$
rucaparib			
ruc1	173	yes	75.9
ruc2	180	yes	43.0
ruc3	173	yes	64.2
ruc4	180	yes	59.1
ruc5	177	yes	151.5
ruc6	179	no	—
rhodamine deriv.			
rhd1	154	yes	69.2
rhd2	141	yes	252.2
rhd3	158	yes	30.9
rhd4	154	yes	144.7

**Table 7:** Affinities for Benchmark Targets. Nanobody designs (**a**) and Protein designs (**b**). Entries in blue correspond to the average of 2 replicate measurements, orange to single measurements. Affinity $K_{D}$ in nM. Expressed designs that do not bind are marked as ◦; lack of expression is marked as ×.

(a) Nanobody designs				
IL-7RA	Insulin	PDGFR	PDL1	TNFα
1.4	890	36	13	◦
23	1300	70	27	◦
26	1400	71	49	◦
38	1500	87	54	◦
120	◦	98	82	◦
150	◦	120	180	◦
240	◦	◦	◦	◦
◦	◦	◦	◦	◦
◦	◦	◦	◦	◦
◦	◦	◦	◦	◦
◦	◦		◦	◦
◦	◦		◦	◦
◦	◦		◦	◦
◦	◦		◦	◦
◦	◦		◦	◦

(b) Protein designs				
IL-7RA	Insulin	PDGFR	PDL1	TNFα
1.9	7.8	0.81	96	◦
66	17	12	310	◦
79	25	23	◦	◦
210	27	110	◦	◦
◦	29	110	◦	◦
◦	33	170	◦	◦
◦	42	260	◦	◦
◦	49	310	◦	◦
◦	54	350	◦	◦
◦	56	350	◦	◦
◦	58	370	◦	◦
◦	140	550	◦	◦
◦	710	◦	◦	◦
◦	730	◦	◦	◦
◦	1400	◦	◦	◦
◦	1400	◦	◦	◦
◦	1600	◦	◦	◦
◦	◦	◦	◦	×
◦	◦	◦	◦	×
◦	◦	◦	◦	×
