# Supplementary material for: BoltzGen: Toward Universal Binder Design
Source: bioRxiv. 2025 Nov 24:2025.11.20.689494. Preprint. [Version 1] doi: 10.1101/2025.11.20.689494 (PMC12697729; doi:10.1101/2025.11.20.689494)
Supplement: 1 [file NIHPP2025.11.20.689494V1-supplement-1.pdf]

bioRxiv preprint doi: https://doi.org/10.1101/2025.11.20.689494; this version posted November 24, 2025. The copyright holder for this preprint (which was not certified by peer review) is the author/funder, who has granted bioRxiv a license to display the preprint in perpetuity. It is made available under aCC-BY 4.0 International license.

Supplementary Material

Appendix Table of contents

A Related Work **37**

B Computational Method Details **37**
B.1 Datasets 37
B.2 Cropping 39
B.3 Training Tasks 40
B.4 Details about Computed Metrics 45

C Additional Computational Results and Details **47**
C.1 Calibrating Filtering Algorithm for Protein-Protein Complexes 47
C.2 Baseline methods 48
C.3 BoltzIF Inverse Folding Model 49
C.4 Motif Scaffolding Benchmark Results 49
C.5 Memorization of Ubiquitin 50

D Learnings from BoltzGenv0 **51**

E Wetlab Experimental Method Details **52**
E.1 Target Selection Process for 10 Hard Adaptyv Bio Targets 52
E.2 BLI and SPR Details for Sections "Designing Nanobodies and Proteins against 9 Novel Targets" and "Designing Nanobodies and Proteins against 5 Benchmark Targets" 53
E.3 Designing Proteins to Bind Bioactive Peptides with Diverse Structures 54
E.4 Designing Peptides to Bind the Disordered Region of NPM1. 57
E.5 Designing Peptides to Bind a Specific Site of RagC and the RagA:RagC Dimer 58
E.6 Designing Nanobodies that Bind Penguinpox and Hemagglutinin 58
E.7 Designing Proteins that Bind to Small Molecules 59
E.8 Designing Antimicrobial Peptides that Inhibit the GyrA to GyrA Interaction 60

bioRxiv preprint doi: https://doi.org/10.1101/2025.11.20.689494; this version posted November 24, 2025. The copyright holder for this preprint (which was not certified by peer review) is the author/funder, who has granted bioRxiv a license to display the preprint in perpetuity. It is made available under aCC-BY 4.0 International license.

A Related Work

Generative Models for Protein Design After initial evidence that structure-generative models can effectively aid protein design Watson et al. [2023], Ingraham et al. [2023], subsequent work has extensively explored this modeling space [Trippe et al., 2022, Anand and Achim, 2022, Luo et al., 2022, Yim et al., 2023b,a, Bose et al., 2024, Liu et al., 2024, Campbell et al., 2024, Wu et al., 2024, Yim et al., 2024]. Further work has expanded these approaches to all-atom target representations [Krishna et al., 2024, Ren et al., 2025] and enzyme design via atomic-motif scaffolding [Ahern et al., 2025]. The latest generation, including BoltzGen and RFDiffusion3 [Butcher et al., 2025], now enables all-atom binder design against targets of almost any modality. The open-source nature of these methods allows the community to scrutinize the results, validate the underlying assumptions, and draw independent conclusions, which ultimately accelerates progress across the field. Several closed-source models also exist [Chai-Discovery et al., 2025, Team et al., 2025, Bio, 2025].

All-Atom Protein Structure Generation Early structure-generative models primarily operated on backbone atoms [Watson et al., 2023], whereas recent approaches perform full all-atom generation [Chu et al., 2024, Qu et al., 2025, Geffner et al., 2025a, Butcher et al., 2025]. A key challenge is that the structure's number of atoms depends on an unknown sequence. Geffner et al. [2025a] circumvent this by encoding side chains in fixed-size latent vectors, but this prevents explicit atom-level reasoning. Chu et al. [2024] represent all possible side chains simultaneously, yet only update one at each denoising step, limiting joint reasoning over residue alternatives. Qu et al. [2025], Butcher et al. [2025], and BoltzGen instead adopt a padded atom14 representation where each designed residue has 14 atoms. While Qu et al. [2025] and Butcher et al. [2025] require a separate predictor for residue types, BoltzGen encodes residue identity directly in its generated geometry, ensuring explicit residue choices throughout denoising.

Inverse Folding Samples from protein structure generative models are typically inverse-folded to obtain sequences compatible with their structures, including in all-atom settings. Most commonly employed methods (but not all [Hsu et al., 2022]) follow the GNN-based formulation of Ingraham et al. [2019], which underpins ProteinMPNN [Dauparas et al., 2022] and its many extensions [?Dauparas et al., 2025]. The BoltzGen inverse folding model is mostly based on these previous works, but is trained on our dataset.

Hallucination-Based Design Hallucination approaches have also shown strong performance in design tasks [Anishchenko et al., 2021, Wicky et al., 2022, Pacesa et al., 2024, Cho et al., 2025a,b, Fang et al., 2025]. Unlike generative modeling approaches, these methods directly optimize sequences using gradients from a folding model.

B Computational Method Details

B.1 Datasets

B.1.1 Training

Our data pipeline builds upon Boltz-2 [Passaro et al., 2025], while adapting the sampling procedure for the task of biomolecular design.

Table 8 summarizes the datasets used for sampling during training, including their sources, sampling cluster types, and associated weights. BoltzGen is primarily trained on entries from the Protein Data Bank (PDB) [Berman et al., 2000] and the AlphaFold Protein Structure Database (AlphaFold DB) [Varadi et al., 2022], while leveraging Boltz-1 distillation [Wohlwend et al., 2025] to enhance performance on underrepresented modalities. For additional information on individual datasets, please see Passaro et al. [2025].

bioRxiv preprint doi: https://doi.org/10.1101/2025.11.20.689494; this version posted November 24, 2025. The copyright holder for this preprint (which was not certified by peer review) is the author/funder, who has granted bioRxiv a license to display the preprint in perpetuity. It is made available under aCC-BY 4.0 International license.

PDB We process every structure in the PDB following a pipeline similar to those previously described in Boltz-2 [Passaro et al., 2025]:

- We use every PDB structure up to the training date cutoff of 06/01/2023. We parse the Biological Assembly 1 from these structures.
- For each polymer chain, we use the reference sequence and align it to the residues available in the structure.
- For ligands, we refer to the CCD dictionary to get the reference ligand and atom composition. We compute up to 10 3D conformers per ligand and sample one at random during training.
- We remove large complexes that are over 7MB or with more than 5000 residues.
- We apply the same filters as AlphaFold3, namely excluding crystallization aids and other non-biologically relevant ligands, removing clashing chains, and filtering out chains with fewer than 4 resolved residues or composed only of unknown residues.
- We compute multiple-sequence alignments for every protein chain (and only protein chains) using ColabFold search. Once monomeric MSAs are produced, we assign a taxonomy ID to every sequence in every MSA using their Uniref100 IDs as reference, if any. The preprocessing of the MSAs is analogous to AlphaFold3.
- We produce template hits for protein chains as described in AlphaFold3, using hmmbuild and hmmsearch on PDB sequences deposited at least 60 days prior to any given query's deposition date.

Distilled datasets We use Boltz-2 distilled datasets, in particular:

- *AlphaFold Database (AFDB) distillation:* In order to construct a protein monomer distillation set, we begin with uniref30 and find the overlap between those sequences and the uniclust multiple sequence alignments provided by OpenFold. We then fetch structures from the AFDB where we impose a minimum global lDDT of 0.5. This procedure results in a monomer distillation of about 5 million proteins.
- *Protein-Ligand distillation*: We construct a dataset of protein-ligand distillation from BindingDB and ChEMBL that were excluded from the main hit-to-lead affinity training set of Boltz-2 [Passaro et al., 2025]. The distillation set was formed by filtering Boltz-1 predictions to examples with a maximum interface predicted distance error (iPDE) ≤ 1.0 and a minimum interface predicted TM-Score (ipTM) ≥ 0.9.
- *RNA distillation*: Following AlphaFold3, we clustered Rfam (v14.9) [Kalvari et al., 2021] using MMSeqs2 [Steinegger and Söding, 2017] with 90% identity and 80% coverage. To form the distillation set, Boltz-1 predictions for cluster representatives are filtered to those where the maximum average predicted distance error (PDE) ≤ 2.0.
- *Protein-DNA distillation*: The protein-DNA distillation data is constructed similarly to AlphaFold3. Using the JASPAR 2024 release (specifically, the CORE collection), we first find transcription factor profiles with matching gene IDs across two high-throughput SELEX datasets [Jolma et al., 2015, Yin et al., 2017]. For each filtered profile, a protein sequence is assigned in two ways: i) using the canonical protein sequence under the profile's Uniprot ID and ii) searching for the sequence in the two SELEX datasets (with matching gene ID) with the highest similarity to the Uniprot sequence. Sequence similarity is calculated using KAlign v2.0, computed as the number of non-gap matches between the two sequences divided by the minimum length of pre-aligned sequences. Unlike AlphaFold3, we did not apply any sequence clustering. To generate binding DNA sequences for each protein sequence, we use the corresponding JASPAR profile's position frequency matrix (PFM) to sample 10 single-stranded motifs. For each distillation example, the inputs include the protein sequence, the single-strand DNA sequence and its corresponding reverse complement. After generating Boltz-1 predictions, we filtered examples to those that satisfied all the following conditions PDE ≤ 2.0, maximum interface predicted distance error (iPDE) ≤ 1.0 and minimum interface predicted TM-Score (ipTM) ≥ 0.7.

bioRxiv preprint doi: https://doi.org/10.1101/2025.11.20.689494; this version posted November 24, 2025. The copyright holder for this preprint (which was not certified by peer review) is the author/funder, who has granted bioRxiv a license to display the preprint in perpetuity. It is made available under aCC-BY 4.0 International license.

Table 8: **Training data composition**

Dataset	Source	Sampling Clusters	Sampling Weight
PDB	experimental	chains & interfaces	0.6
AFDB	AF2 distillation	chains	0.3
Protein-ligand	Boltz-1 distillation	interfaces	0.03
RNA	Boltz-1 distillation	chains	0.04
DNA-protein	Boltz-1 distillation	interfaces	0.03

B.1.2 Structure Prediction Test Set

For structure prediction, the test set in table 20 was constructed following Boltz-2 [Passaro et al., 2025]:

1. Initial release date is between 2024-01-01 and 2024-12-31.
2. Resolution is below 4.5Å.
3. We select all polymer chains that have less than 40% similarity to training polymer chains.
4. We select all interfaces where at least one of the two chains is dissimilar from the training chains.
5. Given these chains and interfaces, we get all the relevant full targets and always predict assembly 1.

We exclude 187 complexes since they do not fit on 40GB GPUs.

B.2 Cropping

Each sampled training entry is randomly cropped. While AlphaFold3 [Abramson et al., 2024] alternates between contiguous, spatial, and interface-spatial cropping, we use the single strategy of Boltz-1 [Wohlwend et al., 2025] tailored to biomolecule design. Algorithm 4 shows how training tokens T are processed: given a center index c determined by the sampling cluster type (Table 8), we iteratively add contiguous fragments of size W from chains nearest to the center until reaching the target crop size L. The maximum crop size is 768 for folding, matching AlphaFold3 and Boltz-2, and 512 for design tasks to accommodate additional memory for fake atoms.

Algorithm 4: CropNeighborhood

Input: tokens T, centre index c, fragment-size W, maximum length L
Output: *cropped* — indices centered on c, grown in W-sized fragments, truncated at L

```
/*order residues by spatial proximity to the center */
ordered ← indices of residues sorted by ‖T[i].x − T[c].x‖;
cropped ← ∅;
for i ∈ ordered do
    chain_members ← { j | T[j].chain_id = T[i].chain_id };
    if |chain_members| ≤ W then
        block ← chain_members ;              // short chain - keep all
    else
        block ← contiguous window in chain_members centered on i,
                expand left/right until |block| ≥ W;
    end
    if |cropped| + |block| > L then
        break ;                              // length budget reached
    end
    cropped ← cropped ∪ block;
end
return cropped (sorted);
```

bioRxiv preprint doi: https://doi.org/10.1101/2025.11.20.689494; this version posted November 24, 2025. The copyright holder for this preprint (which was not certified by peer review) is the author/funder, who has granted bioRxiv a license to display the preprint in perpetuity. It is made available under aCC-BY 4.0 International license.

B.3 Training Tasks

During training, we select different parts of the data sample to be designed. We do this according to different training tasks, which correspond to common use cases such as binder design, or motif scaffolding, as described in Sec. 3.3. The tasks are outlined in Table. 9.

Table 9: **Training Tasks Descriptions**

**Design Task**	**Training Task**	**Description**	**Alg.**
Folding	Folding	No design residues selected	5
Binder Design	Protein Chains	Select a protein chain to be designed	7
	Protein Interfaces	Select residues in a protein chain at the interface with another protein chain	11
	Non-Protein Interfaces	Select residues in a protein chain at the interface with a non-protein chain	10
Motif Scaffolding	Scaffolding	Select all residues in a crop	8
	Motif	Select all residues except a crop	9
Unconditional Design	Standard Protein	Select all protein residues	6

Each task is sampled with a certain probability depending on the data sample. These sampling probabilities are given in Table 10.

Table 10: **Training Tasks Distribution**

**Task**	**Condition**				
		0 Non-Protein		> 0 Non-Protein	
	0 Protein	1 Protein	> 1 Protein	1 Protein	> 1 Protein
Folding (Alg. 5)	1	0.1	0.05	0.05	0.05
Scaffolding (Alg. 9)	0	0.5	0.2	0.2	0.2
Motif (Alg. 8)	0	0.3	0.15	0.15	0.1
Non-Protein Interface (Alg. 10)	0	0	0	0.2	0.05
Standard Protein (Alg. 6)	0	0.1	0.1	0.4	0.1
Protein Interfaces (Alg. 11)	0	0	0.1	0	0.1
Protein Chains (Alg. 7)	0	0	0.4	0	0.4

In addition to selecting which residues will be designed, we also sample other conditioning features, such as binding site specifications. The procedures for each feature are listed in Table 11 along with references to algorithmic descriptions.

Algorithm 5: SELECT_NONE

Input: tokens T
Output: updated T with `T.design_mask` ($1 =$ redesign)
`T.design_mask` $\leftarrow 0$
return T;

bioRxiv preprint doi: https://doi.org/10.1101/2025.11.20.689494; this version posted November 24, 2025. The copyright holder for this preprint (which was not certified by peer review) is the author/funder, who has granted bioRxiv a license to display the preprint in perpetuity. It is made available under aCC-BY 4.0 International license.

Table 11: **Conditioning Inputs Sampling**

Input Feature	Description	Mode	Weight	Alg.
Binding Site	Which residues are part of the binding site, not part of the binding site, or unspecified.	`binding`<br>`not_binding`<br>`both`<br>`none`	0.15<br>0.075<br>0.075<br>0.70	Alg. 12
Pairwise Distances	Which pairwise distances are given as input to the model. Used, for example, to specify the structure of the target.	`all`<br>`uniform`<br>`crops`	0.40<br>0.30<br>0.30	Alg. 13
Secondary Structure	Which residues are part of an alpha helix, beta-sheet, loop, or unspecified.	`all`<br>`uniform`	0.50<br>0.50	Alg. 14

Algorithm 6: SELECT_STANDARD_PROT

```
Input: tokens T
Output: updated T with T.design_mask
foreach residue index i do
    if T[i].is_protein and T[i].is_standard (not modified) then
        T[i].design_mask ← 1;
    end
end
return T;
```

Algorithm 7: SELECT_PROTEIN_CHAINS

```
Input: tokens T
Output: updated T with T.design_mask
chain_ids ← unique{ T[i].chain_id | T[i].is_protein and T[i].is_standard };
chosen_ids ← np.random.choice(chain_ids, size = np.random.randint(1, |chain_ids|));
foreach residue i with T[i].chain_id ∈ chosen_ids do
    T[i].design_mask ← 1;
end
return T;
```

Algorithm 8: SELECT_MOTIF

```
Input: tokens T (one per residue), fragment-size K (desired motif width
Output: updated T with T.design_mask
/* canonical protein residues                                             */
protein_std ← { i | T[i].is_protein and T[i].is_standard };
/* decide maximum motif length                                            */
max_len ← max(np.random.randint(|protein_std|), fragment_size + 1)
/* choose a central residue and gather a contiguous window around that    */
center_token ← np.random.choice(protein_std);
crop_set ← CropNeighborhood(tokens = T, center = center_token, window =
  fragment_size, limit = max_len);
T[crop_set].design_mask ← 1;
return T;
```

bioRxiv preprint doi: https://doi.org/10.1101/2025.11.20.689494; this version posted November 24, 2025. The copyright holder for this preprint (which was not certified by peer review) is the author/funder, who has granted bioRxiv a license to display the preprint in perpetuity. It is made available under aCC-BY 4.0 International license.

Algorithm 9: SELECT_SCAFFOLD

Input: tokens T, fragment-size set K
Output: updated T with `T.design_mask`

/* first select a motif based on SELECT_MOTIF (Alg. 8), then set the remaining standard protein residues as the scaffold to be designed. */

$protein\_std \leftarrow \{\, i \mid T[i].\texttt{is\_protein} \text{ and } T[i].\texttt{is\_standard} \,\};$
$max\_len \leftarrow \max\big(\text{np.random.randint}(|protein\_std|),\ fragment\_size + 1\big)$
$center\_token \leftarrow \text{np.random.choice}(protein\_std);$
$crop\_set \leftarrow \textsc{CropNeighborhood}\big(\text{tokens} = T,\ \text{center} = center\_token,\ \text{window} = fragment\_size,\ \text{limit} = max\_len\big);$

/* design *scaffold* part: all protein-standard residues *outside* the motif */

$T[\,protein\_std \setminus crop\_set\,].\texttt{design\_mask} \leftarrow 1;$
return T;

Algorithm 10: SELECT_NONPROT_INTERFACE

Input: tokens T
Output: updated T with `T.design_mask`

/* pick 1+ non-protein chains as the target and design the k closest standard protein residues at the interface. */

$nonprot\_ids \leftarrow \text{unique}\big\{\, T[i].chain\_id \;\big|\; T[i].\texttt{is\_protein} = \text{False} \,\big\}$
$target\_ids \leftarrow \text{np.random.choice}(nonprot\_ids,\ \text{size} = \text{np.random.randint}(1, |nonprot\_ids|{+}1);$
$candidates \leftarrow \{\, i \mid T[i].\texttt{is\_protein} \text{ and } T[i].\texttt{is\_standard} \,\};$
foreach $i \in candidates$ **do**
  $d[i] \leftarrow \min_{j:T[j].chain\_id \in target\_ids}\Big(\big\|T[i].\texttt{center\_coords} - T[j].\texttt{center\_coords}\big\|_2 + \mathcal{N}(0,\sigma)\Big);$
end
$order \leftarrow candidates$ sorted by d;
$k \leftarrow \text{np.random.randint}(1, |order|{+}1);$
`T[order[:k]].design_mask` $\leftarrow 1;$
return T;

Algorithm 11: SELECT_PROTEIN_INTERFACES

Input: tokens T
Output: updated T

/* Select 1+ protein chains and mark the k standard residues on those chains that lie closest to other protein chains to be designed. */

$prot\_chain\_ids \leftarrow \text{unique}\ \{T[i].chain\_id \mid T[i].\texttt{is\_protein} \text{ and } T[i].\texttt{is\_standard}\}\ ;$
$redesign\_chain\_ids \leftarrow \text{np.random.choice}(prot\_chain\_ids,\ \text{size} = \text{np.random.randint}(1, |prot\_chain\_ids|);$
$Redesign \leftarrow \{\, i \mid T[i].chain\_id \in redesign\_ids \text{ and } T[i].\texttt{is\_standard} \,\};$
$Target \leftarrow \{\, i \mid T[i].chain\_id \notin redesign\_ids \,\};$
foreach $i \in Redesign$ **do**
  $d[i] \leftarrow \min_{j \in \text{Target}}\Big(\big\|T[i].\texttt{center\_coords} - T[j].\texttt{center\_coords}\big\|_2 + \mathcal{N}(0,\sigma)\Big);$
end
$order \leftarrow Redesign$ sorted by d;
$k \leftarrow \text{np.random.randint}(1, |order|{+}1);$
`T[order[:k]].design_mask` $\leftarrow 1;$
return T;

bioRxiv preprint doi: https://doi.org/10.1101/2025.11.20.689494; this version posted November 24, 2025. The copyright holder for this preprint (which was not certified by peer review) is the author/funder, who has granted bioRxiv a license to display the preprint in perpetuity. It is made available under aCC-BY 4.0 International license.

Algorithm 12: SPECIFY_BINDING_SITE

Input: tokens T
Output: updated T with T.`binding_type` set on target residues
$design \leftarrow \{ i \mid T[i].\texttt{design\_mask} = 1 \}$;
$target \leftarrow \{ i \mid T[i].\texttt{design\_mask} = 0 \}$;

`/* compute atom-atom contacts between every target token and all design-token atoms */`
foreach $i \in target$ **do**
    $is\_atomic\_contact[i] \leftarrow \exists\, a \in \text{atoms}(T[i]),\ b \in \text{atoms}(design)$ s.t. $\|a - b\| < 5\,\text{Å}$;
end
$contact\_targets \leftarrow \{ i \in target \mid is\_atomic\_contact[i] = 1 \}$;

$mode \leftarrow$ random.choice([`binding`, `not_binding`, `both`, `none`], p = [0.15, 0.075, 0.075, 0.70]);
if $mode \in \{\texttt{binding}, \texttt{both}\}$ **then**
    $S \leftarrow$ random nonempty subset of $contact\_targets$;
    foreach $i \in S$ **do**
        $T[i]$.`binding_type` $\leftarrow$ BINDING;
    end
end
if $mode \in \{\texttt{not\_binding}, \texttt{both}\}$ **then**
    $U \leftarrow$ random nonempty subset of $(target \setminus contact\_targets)$;
    foreach $i \in U$ **do**
        $T[i]$.`binding_type` $\leftarrow$ NOT_BINDING;
    end
end

return T;

bioRxiv preprint doi: https://doi.org/10.1101/2025.11.20.689494; this version posted November 24, 2025. The copyright holder for this preprint (which was not certified by peer review) is the author/funder, who has granted bioRxiv a license to display the preprint in perpetuity. It is made available under aCC-BY 4.0 International license.

Algorithm 13: SPECIFY_STRUCTURE_GROUPS (pairwise–distance conditioning)

```
Input: tokens T
Output: updated T with T.structure_group set for target residues
/* we set structure_group to drive pairwise-distance conditioning: pairs with group 0 receive no
   distances; pairs whose residues share a nonzero group (≥ 1) have their pairwise distance
   specified                                                                              */
target ← { i | T[i].design_mask = 0 };
chain_ids ← unique T[i].chain_id for i ∈ target;
/* pick one or more target chains to specify structure group                              */
specified ← random subset of chain_ids of size randint[1, |chain_ids|];
/* for each chosen chain, select all tokens or sub-regions to specify the structure       */
subsets ← ∅;
foreach c ∈ specified do
    tokens_c ← { i ∈ target | T[i].chain_id = c };
    mode ← random.choice([all, uniform, crops], p = [0.40, 0.30, 0.30]);
    if mode = all then
        /* specify the entire set of target tokens on the chain                           */
        subsets ← subsets ∪ [tokens_c];
    else if mode = uniform then
        /* split the chain's target tokens into contiguous segments                       */
        m ← randint[1, min(6, |tokens_c|)];
        split tokens_c into m contiguous segments;
        subsets ← subsets ∪ [each segment];
    else crops
        /* take several spatially local crops grown around random centres                 */
        R ← randint[2, 4];
        for r = 1 to R do
            center_token ← random element of tokens not yet chosen in any crop;
            crop_set ← CropNeighborhood(tokens = T, center = center_token, window =
              fragment_size, limit = |tokens not yet chosen in any crop|);
            subsets ← subsets ∪ [crop_set];
        end
    end
end
/* assign frame IDs: sample num_groups and give each subset a random ID in {1, ..., num_groups}
   (group 0 means no distances)                                                           */
num_groups ← randint[1, |subsets|];
foreach S ∈ subsets do
    g ← random choice in {1, ..., num_groups};
    ∀i ∈ S : T[i].structure_group ← g;
end
return T;
```

bioRxiv preprint doi: https://doi.org/10.1101/2025.11.20.689494; this version posted November 24, 2025. The copyright holder for this preprint (which was not certified by peer review) is the author/funder, who has granted bioRxiv a license to display the preprint in perpetuity. It is made available under aCC-BY 4.0 International license.

Algorithm 14: SPECIFY_SECONDARY_STRUCTURE_MASK

```
Input: tokens T
Output: updated T with T.design_ss_mask set for designed residues
/* design_ss_mask controls secondary structure conditioning: 1 means condition on the secondary
   structure for a designed residue                                                          */
designed ← { i | T[i].design_mask = 1 };
/* select a mode for how much SS to reveal                                                   */
mode ← random choice ∈ {all, uniform};
if mode = all then
  | ∀i ∈ designed : T[i].design_ss_mask ← 1;
else uniform
  | /* partition into contiguous intervals; randomly choose to reveal SS or not for each interval
  |    */
  | m ← randint[1, |T|];
  | split the token index range [1..|T|] into m contiguous intervals I_1, ..., I_m;
  | for k = 1 to m do
  |   | with prob 1/2 set T[i].design_ss_mask ← 1 for all i ∈ (designed ∩ I_k);
  | end
end
return T;
```

B.4 Details about Computed Metrics

Table 12 provides a comprehensive reference for all metrics computed by the BoltzGen pipeline.

Table 12: BoltzGen metrics reference.

Metric Name	Description
1 Design quality metrics	
1.1 Predicted structure quality	
`ptm`	Predicted TM-score, measuring overall structure quality (higher is better).
`iptm`	Predicted TM-score across chain pairs, measuring overall complex stability (higher is better).
`design_ptm`	Predicted TM-score for the designed structure, measuring how well the designed structure folds (higher is better).
`design_iptm`	Predicted TM-score for interactions between the entire design chain and target, measuring overall binding interface quality (higher is better).
`design_to_target_iptm`	Predicted TM-score for interactions between only the designed residues (not the full chain) and target, measuring specific binding interface quality (higher is better).
`design_iiptm`	Predicted TM-score for interactions between design residues that are within 8 Å of target atoms and any target residues, measuring interface interaction quality (higher is better).
`design_ptm>[threshold]`	Binary flag for `design_ptm` above threshold (1 = pass, 0 = fail), where threshold is 0.75 or 0.8.
`design_iptm>[threshold]`	Binary flag for design `design_iptm` above threshold (1 = pass, 0 = fail), where threshold is 0.5, 0.6, 0.7, or 0.8.
`interaction_pae`	Predicted aligned error for all design–target interactions (lower is better).

Continued on next page

bioRxiv preprint doi: https://doi.org/10.1101/2025.11.20.689494; this version posted November 24, 2025. The copyright holder for this preprint (which was not certified by peer review) is the author/funder, who has granted bioRxiv a license to display the preprint in perpetuity. It is made available under aCC-BY 4.0 International license.

Metric Name	Description
min_design_to_target_pae	Minimum Predicted Aligned Error (PAE) between any design and target residue pair, indicating the most confidently predicted contact (lower is better).
neg_min_design_to_target_pae	Negative of min_design_to_target_pae for ranking purposes (higher is better).
1.2 Designability / refolding accuracy	
filter_rmsd	Root mean square deviation used for filtering, either backbone RMSD (from_inverse_folded=True) or all-atom RMSD (lower is better).
filter_rmsd_design	RMSD of the designed structure only, used for filtering (lower is better).
designfolding-filter_rmsd	RMSD when refolding the design in isolation (without target), ensuring design stability (lower is better).
2 Interaction metrics	
2.1 Binding interface analysis	
plip_hbonds_refolded	Number of hydrogen bonds between design and target in refolded structure (higher is better).
plip_saltbridge_refolded	Number of salt bridge interactions between design and target in refolded structure (higher is better).
2.2 Binding site adherence	
bindsite_under_[cutoff]rmsd	Fraction of binding site residues within [cutoff] Å of designed residues, where [cutoff] is 3, 4, 5, 6, 7, 8, or 9 (higher is better).
3 Solvent accessibility and hydrophobicity	
3.1 Surface area analysis	
delta_sasa_refolded	Change in solvent accessible surface area when binder is present vs absent, computed on refolded structure (higher indicates better burial).
delta_sasa_original	Change in solvent accessible surface area when binder is present vs absent, computed on original structure (higher indicates better burial).
3.2 Hydrophobicity metrics	
design_chain_hydrophobicity	Hydrophobicity score of the entire designed chain sequence.
design_hydrophobicity	Hydrophobicity score of only the designed residues.
neg_design_hydrophobicity	Negative of design_hydrophobicity for ranking purposes .
design_largest_hydrophobic_ patch_refolded	Area of the largest hydrophobic patch in the refolded design structure (lower is better for solubility).
4 Sequence composition and structure	
4.1 Amino acid composition	
num_design	Number of designed residues in the sequence.
[amino_acid]_fraction	Fraction of specific amino acid residues in the designed sequence.
UNK_fraction	Fraction of unknown (X) residues in the designed sequence (0 preferred to avoid unknown residues).
4.2 Secondary structure	
loop	Fraction of residues in loop conformation (0–1 scale).
helix	Fraction of residues in helical conformation (0–1 scale).
sheet	Fraction of residues in β-sheet conformation (0–1 scale).

Continued on next page

bioRxiv preprint doi: https://doi.org/10.1101/2025.11.20.689494; this version posted November 24, 2025. The copyright holder for this preprint (which was not certified by peer review) is the author/funder, who has granted bioRxiv a license to display the preprint in perpetuity. It is made available under aCC-BY 4.0 International license.

Metric Name	Description
5 Liability analysis	
5.1 Overall liability scores	
`liability_score`	Overall developability score combining all liability assessments (lower is better).
`liability_num_violations`	Total number of liability violations detected in the sequence (lower is better).
`liability_high_severity_violations`	Number of high-severity liability violations (lower is better).
`liability_medium_severity_violations`	Number of medium-severity liability violations (lower is better).
`liability_low_severity_violations`	Number of low-severity liability violations (lower is better).
`liability_violations_summary`	Human-readable summary of all liability violations detected.
`liability_details`	Consolidated details string combining all motif-specific liability information.
5.2 Specific liability motifs	
`liability_[motif]_count`	Number of instances of the specific liability motif found (lower is better). Examples: HydroPatch detects hydrophobic patches like "FILVWY", DPP4 detects cleavage sites like "AP", MetOx detects methionine oxidation sites.
`liability_[motif]_position`	Position of the first occurrence of the motif in the sequence (residue index).
`liability_[motif]_length`	Length of the liability motif in residues.
`liability_[motif]_severity`	Severity score for this motif instance (lower is better). Examples: HydroPatch severity increases with patch size (3+ consecutive hydrophobic residues = high severity), MetOx has moderate severity, DPP4 cleavage sites have high severity.
`liability_[motif]_details`	Specific details about the motif violation.
`liability_[motif]_positions`	All positions where this motif occurs (comma-separated).
`liability_[motif]_num_positions`	Total number of positions where this motif occurs.
`liability_[motif]_global_details`	Global context details for this motif.
`liability_[motif]_avg_severity`	Average severity across all instances of this motif.
7 Affinity prediction (small molecule binders)	
`affinity_probability_binary1`	Probability of binary binding classification (higher is better).
8 Filtering and ranking metrics	
8.1 Aggregated ranking and filtering	
`final_rank`	Final ranking position after quality and diversity optimization (1 = best).
`num_filters_passed`	Number of filter criteria that the design passed.
`pass_filters`	Binary flag indicating whether design passed all filters (1 = pass, 0 = fail).

C Additional Computational Results and Details

C.1 Calibrating Filtering Algorithm for Protein-Protein Complexes

The relative importance of the Boltz-2 confidence metrics and interaction metrics used to rank designs is calibrated on a benchmark of 11,000 validated binders across 11 target proteins, based on data

bioRxiv preprint doi: https://doi.org/10.1101/2025.11.20.689494; this version posted November 24, 2025. The copyright holder for this preprint (which was not certified by peer review) is the author/funder, who has granted bioRxiv a license to display the preprint in perpetuity. It is made available under aCC-BY 4.0 International license.

from Cao et al. [2022]. These weights serve as default values and are manually adjusted for wetlab design experiments based on domain expert feedback.

Binder designs selection benchmark. For each of the 11 targets (InsulinR, FGFR2, EGFR, H3, IL7Ra, PDGFR, SARS-CoV-2 RBD, TGFb, Tie2, TrkA, and VirB8), we sample up to 100 positive examples (i.e., 4μM binders) and fill the remainder up to 1,000 designs with negative examples (i.e., non-binders), resulting in a balanced subset of 11,000 designs.

When exploring different methods to prioritize designs, we optimize the mean enrichment factor at top 25 and top 50 designs. Following Zambaldi et al. [2024], due to the high variance of metric values across targets, we do not directly optimize the mean enrichment score. Instead, we optimize the mean rank across targets, where each target's rank is based on its individual enrichment value. When computing enrichment factors, we normalize by the original binder-to-non-binder ratio from the full dataset, rather than our 11,000-design subset.

Binder designs selection method. In our initial experiments, we trained a decision tree to predict binary binder labels based on the given metric values. However, we find that learning decision thresholds for individual metrics is not optimal, as the best threshold values can be specific to the target protein (for example, optimal interaction count metric thresholds can vary depending on protein size). Instead of using absolute thresholds, we develop a design selection scheme that prioritizes candidates with the best worst-case ranks across all metrics (Algorithm 2). Each metric is assigned a single learnable weight from the set {0, 1, 1.2, 1.5, 2}, where 0 means that the metric is not used. We calibrate these weights by maximizing the enrichment factor on our benchmark of 11,000 experimentally validated binder designs. We do not run this optimization over all metrics but only over a representative subset of non-correlated ones (Figure 22). We include both `design_iiptm` and `neg_min_design_to_target_pae` despite their high correlation, as variations of these two metrics have been shown to be complementary [Zambaldi et al., 2024].

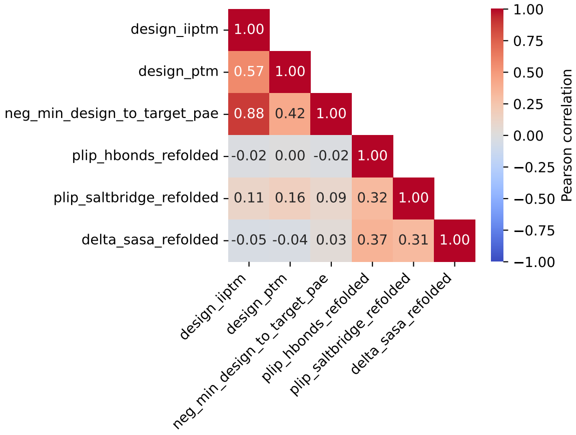

Figure 22: **Final Metrics Used For Binder Design Filtering And Their Pairwise Correlations.** Correlations were calculated on 11,000 BoltzGen-designed binders for 11 targets from our benchmark derived from Cao et al. [2022].

Our enrichment factor optimization, combined with wetlab experimental feedback, yields the following final combination of metrics and weights for Algorithm 2: `design_iiptm`: 1, `design_ptm`: 2, `neg_min_design_to_target_pae`: 1, `plip_hbonds_refolded`: 2, `plip_saltbridge_refolded`: 2, and `delta_sasa_refolded`: 2. When designing small-molecule binders, we slightly modify the weights of Boltz-2 metrics: `design_iiptm`: 1.1, `design_ptm`: 1.1, `neg_min_design_to_target_pae`: 1.1, `plip_hbonds_refolded`: 2, `plip_saltbridge_refolded`: 2, and `delta_sasa_refolded`: 2.

C.2 Baseline methods

RFdiffusion. We employ RFdiffusion [Watson et al., 2023] as a baseline for binder generation, using the official implementation (https://github.com/RosettaCommons/RFdiffusion). We apply the standard settings (`diffuser.T=100`) and reduce the inference noise to improve design quality (`denoiser.noise_scale_ca=0`, `denoiser.noise_scale_frame=0`), following the configuration used in the official binder design example (https://github.com/RosettaCommons/RFdiffusion/blob/main/examples/design_ppi.sh).

We use ProteinMPNN [Dauparas et al., 2022] for inverse folding, as implemented in the official

bioRxiv preprint doi: https://doi.org/10.1101/2025.11.20.689494; this version posted November 24, 2025. The copyright holder for this preprint (which was not certified by peer review) is the author/funder, who has granted bioRxiv a license to display the preprint in perpetuity. It is made available under aCC-BY 4.0 International license.

LigandMPNN [Dauparas et al., 2025] repository (`https://github.com/dauparas/LigandMPNN`). We use the standard checkpoint `proteinmpnn_v_48_020.pt`. Side-chain packing is performed with the following settings: `pack_side_chains=1`, `number_of_packs_per_design=1`, and `pack_with_ligand_context=1`.

RFdiffusionAA. We use RFdiffusion All-Atom (RFdiffusionAA) [Krishna et al., 2024] as a baseline for generating protein binders against small molecules. We employ the official implementation (`https://github.com/baker-laboratory/rf_diffusion_all_atom`) with a standard configuration (`diffuser.T=150`, `inference.ckpt_path=RFDiffusionAA_paper_weights.pt`). Inverse folding is performed using LigandMPNN [Dauparas et al., 2025], as described above for RFdiffusion.

BoltzGen only requires a SMILES representation of the input small molecule and performs cofolding during the design process. In contrast, RFdiffusionAA uses a fixed ligand structure to generate a protein binder. To ensure a fair comparison between the two methods, we generate ligand conformers for RFdiffusionAA using RDKit [Bento et al., 2020]. Specifically, inspired by DiffDock [Corso et al., 2022], we employ the `AllChem.ETKDGv3` algorithm and, if it fails, fall back to initializing random coordinates followed by optimization with `AllChem.MMFFOptimizeMolecule`. We generate a single conformer per input ligand, as we observe that increasing the number of conformers to diversify designs has no significant impact on RFdiffusionAA performance.

C.3 BoltzIF Inverse Folding Model

We verify whether BoltzIF behaves similarly to Protein MPNN (PMPNN) and Soluble (SMPNN) on a set of 64 monomer targets from the PDB. We evaluate each model's ability to inverse fold both native and designed structures. For native ability, we inverse fold the targets themselves and evaluate 50 sequences for each one. For designed ability, we generate 50 binders for each target with BoltzGen and evaluate 1 inverse-folded sequence per design.

Method	Native Backbones		Designed Backbones	
	RMSD < 2.5	Hydrophobic	RMSD < 2.5	Hydrophobic
ProteinMPNN	0.55	1343.30	0.31	1448.65
SolubleMPNN	0.55	1025.72	0.33	1143.31
BoltzIF	0.55	1185.98	0.32	1400.73

Table 13: **Inverse Folding Model Comparison.** "RMSD$<$2.5" denotes the success rate with which designed sequences refold into the inverse folded structure (using Boltz-2). "Hydrophobic" indicates the surface area of the inverse folded protein's largest hydrophobic patch.

Table 13 reports both the backbone designability of the refolded sequences to the original structures as well as the size of the largest hydrophobic patch, which is relevant for protein expressibility. We see that BoltzIF attains the same designabilities as ProteinMPNN and SolubleMPNN and its hydrophobicity scores fall between the two models.

Fig. 23 shows the amino acid distribution over all sequences from each of the models. BoltzIF's residue frequencies mostly fall between that of ProteinMPNN and SolubleMPNN. Likely, the reason for the higher hydrophobicity scores than SolubleMPNN is that it has been trained on crops (hydrophobic cores can be viewed as solvent-facing when it is the surface of a crop).

C.4 Motif Scaffolding Benchmark Results

We ran the following Motif-scaffolding performance benchmark performed in [Geffner et al., 2025b]:

- For each motif scaffolding task, we generate 1000 backbones.

bioRxiv preprint doi: https://doi.org/10.1101/2025.11.20.689494; this version posted November 24, 2025. The copyright holder for this preprint (which was not certified by peer review) is the author/funder, who has granted bioRxiv a license to display the preprint in perpetuity. It is made available under aCC-BY 4.0 International license.

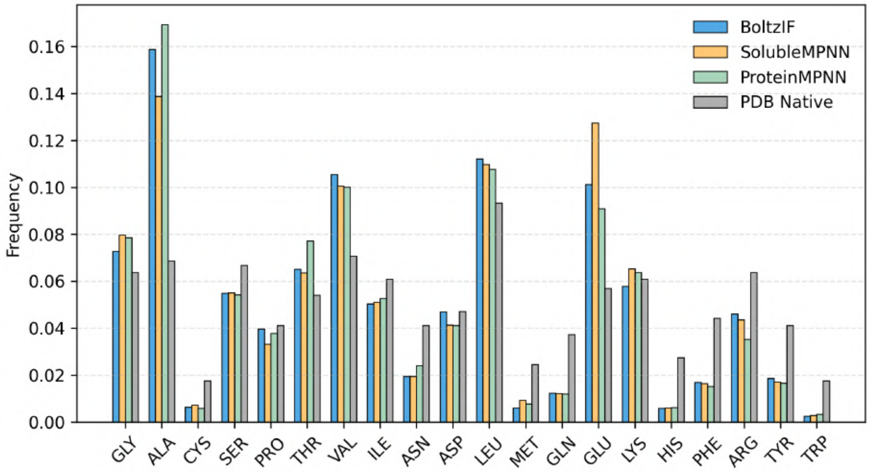

Figure 23: Amino acid distributions when inverse folding BoltzGen's designed binders against 64 monomers in PDB. "PDB Native" denotes the amino acid distribution in our PDB training data.

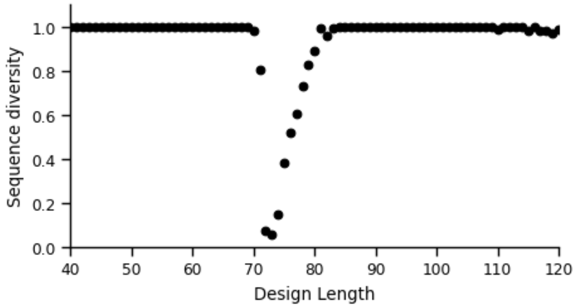

Figure 24: **The Ubiquitin Memorization Issue.** Shown is the sequence diversity (number of unique sequences divided by total number of designs) for designs with varying lengths against 9 targets (33,600 designs). The gap in the 73-76 region stems from BoltzGen's bias toward Ubiquitin in that length range. The bias likely stems from Ubiquitin's overrepresentation in the training data (>1000 entries in the PDB).

- For each backbone, 8 sequences are generated by ProteinMPNN with fixed sequences in the motif region.
- All 8 sequences are refolded via ESMfold and the C_α-RMSD and the motifRMSD are computed between the ground truth and the prediction.
- A backbone is categorized as a success when one of the ProteinMPNN sequences satisfy C_α-RMSD ≤ 2Å, motifRMSD ≤ 1Å, pLDDT ≥ 70, and pAE ≤ 5.
- Hierarchical clustering with single linkage and TM-score threshold 0.6 is performed on all successful backbones to get a clustering to get the final unique successes.

BoltzGen has the highest number of sole best method in 8 tasks (compared to Proteína that wins in 6 tasks).

C.5 Memorization of Ubiquitin

In a few design campaigns, we observed diminished sequence diversity and low filter pass rates for BoltzGen minibinder designs in the 73-76 amino acid length range. This is visualized in an analysis of

bioRxiv preprint doi: https://doi.org/10.1101/2025.11.20.689494; this version posted November 24, 2025. The copyright holder for this preprint (which was not certified by peer review) is the author/funder, who has granted bioRxiv a license to display the preprint in perpetuity. It is made available under aCC-BY 4.0 International license.

Task Name	BoltzGen	Proteina	Genie2	RFDiffusion	FrameFlow
6E6R_short	**83**	56	26	23	25
5TRV_med	**25**	22	23	10	21
5YUI	**36**	5	3	1	1
6EXZ_short	**11**	3	2	1	3
5TRV_short	**6**	1	3	1	1
4JHW	**3**	0	0	0	0
5IUS	**3**	1	1	1	0
1PRW	**2**	1	1	1	1
6E6R_long	289	**713**	415	381	110
6EXZ_long	59	290	326	167	**403**
6E6R_medium	164	**417**	272	151	99
1YCR	102	**249**	134	7	149
5TRV_long	155	**179**	97	23	77
4ZYP	1	**11**	3	6	4
6EXZ_med	32	43	54	25	**110**
7MRX_128	64	51	27	**66**	35
7MRX_85	22	**31**	23	13	22
3IXT	10	8	**14**	3	8
5TPN	3	4	**8**	5	6
7MRX_60	4	2	**5**	1	1
1QJG	0	3	5	1	**18**
1BCF	1	1	1	1	1
5WN9	2	2	1	0	**3**
2KL8	1	1	1	1	1

Table 14: Number of unique successes on the RFDiffusion benchmark for BoltzGen and 4 other methods, for 1000 backbones.

33,600 designs against 5 protein and 4 small molecule targets in Figure 24. Inspection of the sequences revealed that the pipeline (backbone design followed by inverse folding) is frequently recapitulating the sequence for ubiquitin in this length range. For example, in this analysis all designs of length 73 (n=156) had >97% sequence identity to "MQIFVKTLTGKTITLEVEPSDTIENVKAKIQDKEGIPPDQQRLIFAGKQLEDGRTLSDYNIQKESTLHLVLRL".

Likely, this arises since this sequence is present, often in complex, in >1000 entries in the PDB. In future versions of the model, we plan to down-sample this interaction during training.

D Learnings from BoltzGenv0

Template bug. A previous version of BoltzGen, which we term BoltzGenv0, had a serious flaw resulting in close-to-random ranking and filtering. We can judge the impact of the bug based on the nanobody design results in Section 2.7, where, with the current version of BoltzGen, we obtain a 1/7 hit rate for the Penguinpox target and a 7/7 hit rate against hemagglutinin. BoltzGenv0's hit rates were 0 for both targets. The same failure case occurred for an attempt to design helicons against a pMHC complex using BoltzGenv0.

The nature of this bug was in our handling of which residues are considered to be part of the target and which part of the design. In cases where the designed binder contains fixed residues, such as when designing helicons or nanobodies, BoltzGenv0 considered the fixed residues of the designs as being part of the target. The implications of this are that their relative position with respect to the target is provided in the refolding step via the templates that we employ for the targets. Thus, the resulting structure prediction is bound to recapitulate the generated structure, without providing any filtering power that enriches for binders. Furthermore, metrics such as the minimum interaction pAE and the ipTM would be influenced by the fixed residues that are in the design. For instance, in the overwhelming

bioRxiv preprint doi: https://doi.org/10.1101/2025.11.20.689494; this version posted November 24, 2025. The copyright holder for this preprint (which was not certified by peer review) is the author/funder, who has granted bioRxiv a license to display the preprint in perpetuity. It is made available under aCC-BY 4.0 International license.

majority of cases, the minimum interaction pAE would not correspond to an interaction between the design and the target, but rather an interaction between a designed residue and a fixed residue in the designed binder. Hence, these scores also do not provide any filtering power for BoltzGenv0.

Designfolding. Another improvement in BoltzGen over BoltzGenv0 is its "designfolding" step. In this step, we additionally refold the design in the absence of the target and compute the RMSD to the design in the generated structure. This serves as a proxy to assess whether the binder could attain the designed structure by itself, which we use to filter out designs that are likely to require a significant conformational change upon binding or do not express since they do not fold into a stable structure by themselves.

We introduced this improvement after a protein-protein binder design attempt where all 12 of BoltzGenv0's designs failed to express. These designs would often partially or completely "envelop" the target and require a large conformational change to bind.

E Wetlab Experimental Method Details

E.1 Target Selection Process for 10 Hard Adaptyv Bio Targets

For assembling a panel of hard protein targets for Adaptyv Bio to test experimentally, we use the following criterion:

1. **PDB Monomer** — The biological assembly has to be a monomer with exactly one protein polymer instance (`oligomeric_state = Monomer`, `polymer_entity_instance_count_protein = 1`).
2. **Monomer-only sequence cluster** — Each chain is either a singleton or a member of a sequence cluster (30% identity threshold, `mmseqs easy-cluster` with `-min-seq-id 0.30` and `-c 0.0`) in which *every* member is a PDB monomer (satisfies Condition 1).
3. **Catalog availability** — Must be available in the *Sino Biological* catalog (via mapping to a Swiss-Prot accession listed there).

With this selection method, we aim to ensure that each protein we keep is a **monomeric PDB entry** that has no close sequence homolog (MMseqs2 sequence identity $\geq$ 30%) anywhere in the PDB that appears in a multimeric or ligand-bound assembly. This makes the targets genuinely "hard" for our binder design: the model has not seen a closely related protein in a bound context during training.

We verify our target's sequence identities $<$30% to any non-monomeric protein in PDB as follows. For each target sequence, we ran MMseqs2 easy-search against non-monomer PDBs and keep the top identity hit that passes our coverage filter:

```
mmseqs easy-search queries.fa nonmonomer_db out.m8 tmp \
  --threads 32 \
  --min-seq-id 0.0 \
  --alignment-mode 3 \
  -e 1e5 -s 9.5 \
  --prefilter-mode 2 \
  --cov-mode 2 -c 0.9 \
```

- **Coverage policy.** We require *near-global coverage on the target* via `-cov-mode 2 -c 0.9`, i.e.

$$\text{tcov} = \frac{\text{aligned residues}}{\text{target length}} \geq 0.9.$$

  This enforces that the match spans (almost) the entire *target* chain. We initially used `-c 1.0`, but very long targets often fail to align end-to-end stably; we therefore relaxed to 0.9.

bioRxiv preprint doi: https://doi.org/10.1101/2025.11.20.689494; this version posted November 24, 2025. The copyright holder for this preprint (which was not certified by peer review) is the author/funder, who has granted bioRxiv a license to display the preprint in perpetuity. It is made available under aCC-BY 4.0 International license.

- **Zeros in the table.** Some entries appear as 0.0% (e.g., **1JQD**, **2A1X**). This indicates that no hit met the near-global coverage threshold, not that a full-length alignment with 0% identity was found. These two sequences are also the longest among the ten (292 and 308 aa), making full-length alignment to known non-monomeric chains particularly hard under tcov ≥ 0.9.

Table 15: **Maximum sequence identity** (against non-monomeric PDB chains) for the selected targets.

Target	Max sequence identity (%)
1G13	25.4
1JQD	0.0
1NB0	24.6
2A1X	0.0
2PNY	23.0
3APU	19.8
3CH4	22.7
3QKG	27.9
7AAH	24.3

E.2 BLI and SPR Details for Sections "Designing Nanobodies and Proteins against 9 Novel Targets" and "Designing Nanobodies and Proteins against 5 Benchmark Targets"

The binding affinity assays were carried out by the contract research organization Adaptyv Bio.

Biolayer Interferometry (BLI) affinity characterization Ligand constructs were designed by reverse-translating target protein sequences and optimizing codon usage for expression in a prokaryotic cell-free system. A C-terminal assay tag was included to facilitate capture on biosensors. Gene fragments (Twist Bioscience) were assembled using NEBuilder HiFi DNA Assembly (NEB) in 2 μL reactions. Assembly products were validated via capillary electrophoresis (Agilent ZAG DNA Analyzer) and quantified using the Qubit dsDNA assay (Invitrogen).

Proteins were expressed in 8 μL reactions using an optimized in vitro transcription/translation system supplemented with 4 nM DNA template. Reactions were incubated at 37 °C for 8 hours. After expression, total protein levels were measured using an affinity-based detection assay and normalized across samples prior to binding analysis.

BLI experiments were carried out using a Gator Bio instrument with Strep-Tactin XT biosensors. Twin-Strep-tagged ligands were captured on the biosensors using the following protocol:

- **Baseline 1:** 120 s in running buffer
- **Ligand loading:** 120 s (target shift 0.5–1.0 nm)
- **Baseline 2:** 200 s in running buffer

The running buffer consisted of 50 mM HEPES, 100 mM NaCl, and 0.5% Triton X-100 at pH 7.4. All steps were performed at 25 °C with data collected at 5 Hz. Binding was assessed using a multi-cycle format with four antigen concentrations (30–1000 nM, half-log dilution series). Each kinetic cycle consisted of a 220 s association phase in antigen solution followed by a 240 s dissociation phase in running buffer. After each cycle, biosensors were regenerated with 10 mM glycine-HCl (pH 1.5) applied five times for 10 s each, followed by a wash step in running buffer to restore baseline. Buffer-only and non-binding ligand controls were used for reference subtraction and signal drift correction.

bioRxiv preprint doi: https://doi.org/10.1101/2025.11.20.689494; this version posted November 24, 2025. The copyright holder for this preprint (which was not certified by peer review) is the author/funder, who has granted bioRxiv a license to display the preprint in perpetuity. It is made available under aCC-BY 4.0 International license.

Surface Plasmon Resonance (SPR) affinity characterization SPR measurements were performed on a Carterra LSA XT system. DNA constructs encoding ligands with C-terminal Twin-Strep tags were synthesized (Twist Bioscience), assembled using NEBuilder HiFi DNA Assembly, and validated using capillary electrophoresis and Qubit fluorometry. Proteins were expressed in a prokaryotic in vitro translation system and normalized post-expression using an affinity-based quantification assay. Sensor chip surfaces were functionalized by covalently attaching Strep-Tactin XT to a carboxymethylated surface using EDC/NHS coupling. The chip preparation procedure included:

- **Conditioning:** 50 mM NaOH
- **Activation:** EDC/NHS solution
- **Capture:** Strep-Tactin XT (50 μg/mL in 10 mM sodium acetate, pH 4.5)
- **Quenching:** 1 M ethanolamine hydrochloride, pH 8.5
- **Wash:** 0.1 M sodium borate, 1 M NaCl, pH 9.0

Twin-Strep-tagged ligands were captured on the chip using a 96-channel printhead under bidirectional flow for 750 s, followed by a 600 s baseline step in running buffer. Antigens were diluted in running buffer (10 mM HEPES, 150 mM NaCl, 3 mM EDTA, 0.05% Tween-20, pH 7.4) and injected at seven concentrations (1–1000 nM, half-log dilution series) in a single-cycle kinetic format. Each cycle consisted of a 60 s baseline step in running buffer, a 300 s antigen association phase, and a 600 s dissociation phase in running buffer. After the completion of each injection series, the chip was regenerated with 10 mM glycine-HCl (pH 1.5) for 5 minutes, followed by a 20-minute wash in running buffer.

Data analysis and binder classification Sensorgrams from both BLI and SPR assays were analyzed using Adaptyv Fitting software. Preprocessing included trimming to relevant kinetic phases (association and dissociation), correcting for signal jumps at buffer transitions, aligning phases, and subtracting signals from both baseline and reference channels.

Data were fit to a 1:1 Langmuir binding model using a global fitting approach across all antigen concentrations. If global fits were not feasible, alternative fitting strategies—such as dissociation-only or slope-based methods—were employed. In cases where individual curve fits could not be achieved, group-level models (e.g., equilibrium, flat, or linear) were used to approximate binding behavior.

Final kinetic parameters (k_{on}, k_{off}, and K_D) were determined based on the best available fit. Under global fitting, k_{off} and K_D were estimated directly, with k_{on} calculated as k_{off}/K_D. Ligands were labeled as **binders (True)** or **non-binders (False)** based on the presence of quantifiable sensorgrams and successful kinetic model fitting. In cases where ligands generated a large signal during the association phase (at least 300% greater than the negative control) but could not be reliably fit, binding classification was assigned based on the observed magnitude of the shift.

E.3 Designing Proteins to Bind Bioactive Peptides with Diverse Structures

Experiments by A. Katherine Hatstat, Angelika Arada, Nam Hyeong Kim, Ethel Tackie-Yarboi, Dylan Boselli, Lee Schnaider, and William F. DeGrado

Visual Inspection The top 100 computationally ranked designs were manually examined in PyMOL. Visual inspection criteria included: (i) extent of peptide burial within the binding pocket, (ii) number and geometry of hydrogen bonds between binder and target peptide, (iii) overall packing density and complementarity at the binding interface, and (iv) internal packing quality of the apo binder in the absence of the target peptide. The top 6 designs exhibiting consistent burial, multiple well-oriented hydrogen bonds, and tightly packed interfaces were prioritized for experimental characterization.

Solid phase peptide synthesis and purification Protegrin-1 was purchased from MedChemExpress (catalog #HY-P1633)/ Melittin and Indolicidin were synthesized following the procedure below.

bioRxiv preprint doi: https://doi.org/10.1101/2025.11.20.689494; this version posted November 24, 2025. The copyright holder for this preprint (which was not certified by peer review) is the author/funder, who has granted bioRxiv a license to display the preprint in perpetuity. It is made available under aCC-BY 4.0 International license.

Melittin and Indolicidin were synthesized on a Biotage Initiator Alstra microwave synthesizer using standard Fmoc solid-phase peptide synthesis on a TentaGel S Ram resin. Resin (417 mg, 0.24 mmol) was swollen in DMF for 10-15 mins prior to synthesis. The general synthetic steps included: (a) Fmoc deprotection with 20% (v/v) piperidine in DMF, (b) resin washing (3x, DMF), (c) amino acid (0.50 mmol) coupling with DIPEA (0.50 mmol) and HCTU (0.50 mmol) for 5 min at 75 °C, (d) resin washing (3x, DMF), and (e) repeat deprotection/coupling until sequence completion.

The peptides were globally deprotected in a 10 mL solution of TFA:H2O:TIPS (95:2.5:2.5) for 3 h. The solution was then filtered, with filtrate concentrated under the flow of nitrogen. The concentrate was precipitated in cold diethyl ether (40 mL), centrifuged, and the pellet dissolved in 5 mL H2O:ACN (1:1, 0.1% TFA).

Crude peptides were purified by reverse-phase HPLC on a C18 column using H2O/ACN (0.1% TFA) at a 10 mL/min gradient of 5-100% ACN (0.1% TFA) for 50 min. Pure fractions were identified by analytical HPLC and MALDI-TOF, pooled, and lyophilized. The final products were white powders with ≥95% purity.

Protein expression and purification Codon-optimized genes encoding the designed candidate proteins with an N-terminal 6×His tag and a TEV protease cleavage site (HHHHHHENLYFQS) were synthesized and obtained from Twist Bioscience. To facilitate Gibson assembly into the pET-28a(+) vector, short sequences were added at the 5' end (CTCTAGAAATAATTTTGTTTAACTTTAA-GAAGGAGATATACC) and 3' end (GATCCGGCTGCTAACAAAGCCCGAAAG) of each gene. The recombinant plasmids were transformed into Escherichia coli strain E. cloni BL21(DE3). A single colony was picked from an LB agar plate and inoculated into LB medium supplemented with kanamycin (50 μg/mL) for overnight growth. The culture was then transferred into 200 mL of TB medium containing kanamycin (50 μg/mL) and incubated at 37 °C until reaching an OD600 of 0.6 - 0.8. Protein expression was induced with 0.5 mM isopropyl β-D-1-thiogalactopyranoside (IPTG), and cultures were incubated overnight at 30 °C. Cells were harvested by centrifugation and resuspended in 25 mL PBS buffer (10 mM Na2HPO4, 1.8 mM KH2PO4, 2.7 mM KCl, 137 mM NaCl, pH 7.4) supplemented with 20 mM imidazole. Cells were lysed by ultrasonication (Sonic Dismembrator Model 500, Fisher Scientific), and the lysate was clarified by centrifugation (35,000g, 30 min). The supernatant was loaded onto a gravity column containing Ni-NTA agarose resin (HisPur, Thermo Fisher, 1.0 mL or 3.0 mL). The resin was washed with three column volumes (CVs) of PBS buffer containing 20 mM imidazole, and bound proteins were eluted with 7 mL PBS buffer containing 250 mM imidazole. The eluted proteins were concentrated and subjected to three rounds of buffer exchange with PBS buffer using a 15 mL, 10 kDa cutoff centrifugal filter unit (EMD Millipore).

Circular dichroism Protein samples were prepared at 10 μM in sterile filtered 10 mM sodium phosphate with 50 mM NaCl, pH 7.4. A280 was measured via UV-Vis spectroscopy in a 0.1 mm Quartz cuvette, and protein concentration was calculated from A280 via Beer's law using the extinction coefficient calculated from protein sequence via ExPasy ProtParam. Circular dichroism measurements were performed on a Jasco J-810 spectropolarimeter. Spectra were collected from 200-250nm in continuous scanning mode at 50 nm/min and 1nm band width with six accumulations per sample. CD spectra were converted from millidegrees to molar ellipticity using the equation $m \cdot M/(10 \cdot L \cdot C)$ where C is concentration in g/L (derived from A280 signal in UV-Vis experiments), M is the average molecular weight (g/mol) and L is the path length of the cell.

Analytical size exclusion chromatography The oligomeric state of binder samples was assessed via analytical size exclusion chromatography using a Superdex 75 5/150 analytical gel filtration column (Cytiva) on an AKTA FPLC. Samples (50 μL) were prepared at 100 μM in sterile filtered 1X Phosphate Buffered Saline (PBS), pH 7.4 and centrifuged in a microfuge at 21,000g for 15 minutes before loading onto the FPLC. Chromatography runs were conducted at 0.2 mL/min for 1.2 column volumes and absorbance was measured at 220 and 280 nm. For measurement of protein:peptide complexes, protein and peptide were mixed at a 1:1 ratio (50 μM each) and incubated overnight at 4 °C. After equilibration to room temperature, samples were centrifuged at 21,000g for 15 minutes before loading onto the FPLC.

bioRxiv preprint doi: https://doi.org/10.1101/2025.11.20.689494; this version posted November 24, 2025. The copyright holder for this preprint (which was not certified by peer review) is the author/funder, who has granted bioRxiv a license to display the preprint in perpetuity. It is made available under aCC-BY 4.0 International license.

Change in intrinsic tryptophan fluorescence for *in vitro* assessment of peptide binding *In vitro* binding was assessed via tryptophan quenching in which either peptide or protein was held constant with the other binding partner varied depending on which species contained tryptophan residues. All peptides were solubilized in DMSO at > 1mg/mL prior to dilution into sterile filtered 1X PBS, pH 7.4 for binding experiments. All binding assays were conducted in PBS in non-binding 96-well half-area black plates (Corning 3686) and tryptophan quenching was measured as endpoint fluorescence intensity measurements ($\lambda_{ex} = 295$ nm, $\lambda_{em} = 330$ nm) in a BioTek Synergy Neo-2 multi-mode plate reader. For melittin and indolicidin, which both contain tryptophan residues, assays were conducted with constant [peptide], and binder sequences were designed to exclude tryptophan. [Melittin] was fixed at 10 μM and [melittin binder] was varied from 0 to 40 μM. [Indolicidin] was initially fixed at 10 μM, with [binder] varying from 0-20 μM. For subsequent global fitting experiments, [Indolicidin] was held constant at either 5, 7.5 or 10 μM and [binder] was varied from 0-25μM. Samples were prepared in triplicate and incubated overnight at 4 °C. After equilibrating to room temperature for 30 minutes, tryptophan fluorescence was measured as described. As a control, a gradient of [binder] without peptide was included for background subtraction. For all samples, the A295 and A330 of the binder was below 0.1; thus, protein fluorescence was subtracted as background instead of being treated with inner filter effect correction. For protegrin, which contains no tryptophan residues, [binder] was held constant while [peptide] was varied. All protegrin binder designs contain at least one tryptophan. For initial experiments, [protegrin] was held constant at 5 μM and [binder] was varied from 0-30μM. Samples were prepared in triplicate and incubated at room temperature for 3h before tryptophan fluorescence was measured. Here, a gradient of [protegrin] without binder was included for background subtraction.

Binding was fit with the following quadratic binding equation in Prism (GraphPad): $Y = M + ((Q - M)/(2 \cdot P)) \cdot ((1/K) + X + P - \sqrt{((1/K) + X + P)^2 - 4 * P * X})$ where M = fully unbound signal (baseline signal), Q = fully bound (saturation) signal, P = concentration of fixed species, X = ligand concentration, Y = observed fluorescence intensity, and K = association constant. M, Q, and K were not constrained, and P was fixed. For global fitting, K was shared for all datasets.

paragraphSurface plasmon resonance for validation of indo4-indolicidin binding The binding of the highest affinity peptide/binder pair was further validated by surface plasmon resonance (SPR). SPR was performed on a Bruker SPR-24 Pro Instrument using an NTA derivatied SPR chip (SPR sensor prism NiHC1000M; Xantec bioanalytics). The surface was preconditioned with 350mM ethylenediaminetetraacetic acid (EDTA) and running buffer (10 mM HEPES pH 7.4, 150 mM NaCl, 50 µM EDTA, 0.05% Tween-20) prior to loading with 5mM Ni2+. Hexahistidine tagged indo4 binder was immobilized on the surface prior to exposure to analyte (indolicidin). Indolicidin was solubilized in water to $\tilde{4}$mg/mL to afford a stock solution. From the stock solution, a concentration gradient of 0-20μM indolidicin was prepared in running buffer. The analyte solutions were flowed over the immobilized protein surface for 80 seconds at 25 μL/min flow rate from low to high concentration and 120 second dissociation time. Blank (running buffer only) injections interspersed between the analyte injections to confirm that analyte was dissociating between injections. Following the cycle of injections, binding affinity was calculated by plotting the pre-injection stop point signal (RU) versus protein concentration. High concentration samples were omitted because of bulk shift from buffer mismatch from preparation of the samples from stock solution solubilized in water. Affinity was calculated via a Langmuir fit of the response units (RU) at the pre-injection stop point.

Bacterial growth assays to measure neutralization of antimicrobial activity Minimum inhibitory concentrations (MICs) of melittin, indolicidin, and protegrin-1 were determined against Bacillus subtilis (ATCC 23857). Peptides were prepared at 100 μg/mL and serially diluted 2-fold in Mueller Hinton Broth (MHB). A glycerol stock of B. subtilis was inoculated into 10 mL MHB and grown overnight at 37°C. The following day, 100 µL of starter culture was added to 10 mL MHB and grown to an OD600 of 0.6–0.8, then diluted to OD600 = 0.001 and added to the peptide dilutions in a 96-well non-treated cell culture plate (Gen-Clone 25-104). Absorbance at 600 nm was measured using a BioTek Synergy Neo-2 plate reader at 37°C with cycles of 7 min shaking and 3 min rest for 15 hours. The MIC was defined as the lowest peptide concentration that completely inhibited growth, and was 1.1 μM, 1.70 μM, and 1.16 μM for melittin, indolicidin, and protegrin-1, respectively.

For neutralization assays, peptides were held constant at their respective MICs, and protein binders were serially diluted 2-fold starting from 40X its target peptide MIC. Peptide was added to the protein

bioRxiv preprint doi: https://doi.org/10.1101/2025.11.20.689494; this version posted November 24, 2025. The copyright holder for this preprint (which was not certified by peer review) is the author/funder, who has granted bioRxiv a license to display the preprint in perpetuity. It is made available under aCC-BY 4.0 International license.

binders, transferred to a 96-well plate, and then the diluted B. subtilis culture prepared as described above was added. Samples were prepared in triplicates. Absorbance at 600 nm was measured as previously described, with the exception that the indolicidin assays were conducted for 7.5 hours instead of 15 hours to account for peptide degradation. For the protegrin binders, protein and peptide dilutions were incubated overnight at 4°C before measuring absorbance. % neutralization was calculated with the following equation: % neutralization $= ((A_{\text{obs}} - A_{\text{min}})/(A_{\text{max}} - A_{\text{min}}))cdot100$ where A_{obs} is the observed endpoint A600 for the varying protein concentrations, A_{min} is the observed endpoint A600 of the peptide and bacteria only control, and A_{max} is the observed endpoint A600 of the bacteria only control.

Hemolysis assays Sheep red blood cells (25 mL) were transferred into a 50 mL conical tube and centrifuged at 500 x g for 5 min. The plasma layer was aspirated, leaving the pellet. The cells were resuspended in 150 mM NaCl solution to 25 mL, with gentle inversion and centrifugation (500 x g, 5 min). The supernatant was aspirated, and washed once more with 150 mM NaCl solution. The pellet was then resuspended in 1X PBS (pH 7.4), centrifuged (500 x g, 5 min), and aspirated. The pellet after the PBS wash, was then resuspended in 1X PBS to 25 mL and stored at 4 °C. For hemolysis assessment, 190 μL of RBC (1:100 in 1X PBS) was added per well of a 96-well plate, followed by the addition of 10 μL of either 1X PBS, 20% Triton X-100, or melittin. Cells were treated with serial dilutions of melittin (0.08 to 10 μM final). The plate was incubated at 37 °C with gently shaking for 1 h, followed by centrifugation (500 x g, 5 min). From each well, 100 μL of supernatant was transferred to a fresh plate ensuring pellets were undisturbed. Absorbance was measured at 400 nm using a microplate reader (SpectraMax M5). Values were normalized to PBS and Triton X-100 controls (N= 4, performed in duplicate). To assess melittin's hemolytic activity in the presence of its binders, 10 μL of melittin (1.2 μM final) was added to wells of a 96-well plate, followed by serial dilution of protein (0.05 μM to 6 μM final). This was incubated for 1h at room temperature. Then, 180 μL of red blood cells (1:100 in 1X PBS) was added to each well and incubated at 37 °C for 1 h. After centrifugation, 100 μL of supernatants were transferred to a fresh plate and absorbance measured at 400 nm. Absorbance values were normalized to PBS and melittin only (1.2 μM) controls (N= 4, performed in duplicate).

E.4 Designing Peptides to Bind the Disordered Region of NPM1.

Experiments by Yaotian Zhang, and Denes Hnisz

pRK5-msfGFP-NPM1binder plasmids were constructed by amplifying msfGFP from pRK5_msfGFP-HMGB1-Shuffled 1 (Addgene #237650) (PMID: 40468084). The NPM1binder sequences were inserted at the C-terminus of msfGFP through primer sequences. The amplicons were assembled into AgeI + XbaI-digested pRK5 backbone (Addgene #194548) (PMID: 36755093) using the NEBuilder HiFi DNA assembly master mix.

Cell culture Cells were cultured under standard conditions (37 °C and 5% CO_2) in sterile, TC-treated, non-pyrogenic, polystyrene tissue culture dishes (Corning). U2-OS (ATCC, HTB-96) cells were cultured in DMEM GlutaMAX (Gibco, 31966047). The culture medium included 10% FBS (Gibco, 10438-026) and 100 U ml^{-1} penicillin–streptomycin (Gibco, 15140148). All cell lines tested negative for mycoplasma using the LookOut Mycoplasma PCR Detection Kit (Sigma-Aldrich, MP0035) or the PCR Mycoplasma Test Kit II (Applichem, A8994). Mycoplasma testing was performed on 0.2–1 ml of culture medium taken from tissue culture dishes containing confluent monolayers of cells, on a routine basis at least twice a year.

Live-cell imaging All live-cell imaging experiments were performed using the LSM880 Airyscan microscope equipped with a Plan-Apochromat 63×/1.40 oil differential interference contrast objective, while incubating cells at 37 °C and 5% CO_2. Cells were seeded onto eight-well chamber slides (Ibidi, 80826-90) at 40,000 cells per well, transfected 24 h later, and imaged 24 h after transfection. U2OS cells were transfected using FuGENE HD according to the manufacturer's instructions. Hoechst 33342 (0.2 µg ml^{-1}, Thermo Fisher Scientific, 62249) was added to the cell culture medium for nuclear staining.

bioRxiv preprint doi: https://doi.org/10.1101/2025.11.20.689494; this version posted November 24, 2025. The copyright holder for this preprint (which was not certified by peer review) is the author/funder, who has granted bioRxiv a license to display the preprint in perpetuity. It is made available under aCC-BY 4.0 International license.

Live-cell imaging For immunofluorescence experiments, U2OS cells were seeded on eight-well chamber slides (Ibidi, 80826-90) at 40,000 cells per well, transfected 24 h later, and fixed 24 h after transfection with 4% PFA in PBS for 10 min. Cells were permeabilized with 0.5% Triton X-100 (Thermo Fisher Scientific, 85111) in PBS for 30 min, incubated in blocking buffer containing 1% BSA (BSA Fraction V, Gibco, 15260037) and 0.1% Triton X-100 in PBS for 1 h, and stained with primary antibodies at room temperature for 1 h with gentle rotation. Slides were washed five times with blocking buffer, incubated with secondary antibodies (AlexaFluor 594 donkey anti-mouse antibody, Jackson ImmunoResearch, 715-585-150; and AlexaFluor 594 donkey anti-mouse antibody, Jackson ImmunoResearch, 711-605-152; 1:1,000) in blocking buffer for 1 h at room temperature, washed twice with blocking buffer, stained with 0.5 µg ml^{-1} DAPI in PBS (Invitrogen, D1306), and washed three times with PBS. The following primary antibodies were used: NPM1 (B23) (Santa Cruz, sc-271737, 1:100) and SURF6 (Abcam, ab221990, 1:1000). Imaging was performed using the LSM880 Airyscan microscope equipped with a Plan-Apochromat 63×/1.40 oil differential interference contrast objective.

E.5 Designing Peptides to Bind a Specific Site of RagC and the RagA:RagC Dimer

Experiments by Shamayeeta Ray, Jonathan T. Goldstein, and David M. Sabatini.

Expression and purification of the Rag A: Rag C GTPase heterodimer E.coli LOBSTR [Andersen et al., 2013] cells carrying a pETDuet-1 vector encoding codon optimized, C-terminally His-tagged RagA with a mutation (T21N) that favors the GDP loaded state[Shen et al., 2017, Kim et al., 2008, Yang et al., 2020] and tagless wildtype (WT) RagC in its state (Addgene: 99664), were grown in Terrific broth (TB) and protein expression induced with an overnight IPTG treatment at 18°C b. The purification follows a protocol described previously[Shen et al., 2017]. The complex was purified using Ni-NTA affinity chromatography followed by ion-exchange chromatography (IEX) using a Capto HisRes Q anion-exchange column and then size-exclusion chromatography (SEC) using a Superdex200 column. The protein corresponding to the heterodimer was concentrated in a final buffer containing 50 mM HEPES (pH7.5), 100 mM NaCl and 2 mM MgCl2 and used for SPR studies.

Binding studies of peptides to the Rag GTPase heterodimer using a high-throughput SPR instrument Surface plasmon resonance (SPR) experiments were performed on a Cytiva Biacore 8K instrument. 0.2 μM of the Rag GTPase heterodimer with the His-tag on RagA was immobilized on a Biacore NTA sensor chip using a Ni-NTA-Histag immobilization technique. All the peptides, at a concentration range from 0-100 μM were flown over the protein-bound NTA sensor chip as 'analyte' and their binding responses were recorded. The Rag GTPase heterodimer was first loaded with 1 μM GDP after immobilization prior to each peptide run. The protein, GDP, and the peptide samples were prepared in a buffer containing 50 mM HEPES (pH7.5), 100 mM NaCl and 2 mM MgCl2. Rag GTPase heterodimer was immobilized on the NTA sensor chip for 200 sec and all the runs were performed at 25°C. For each peptide run at each concentration, the association and dissociation times were 120 and 300 sec, respectively. The sensor chip was regenerated after each run using 0.35 M EDTA and subsequently reused throughout the entire run. Association and dissociation kinetics along with binding affinities were analyzed using the Biacore™ Insight Software. Each sensogram corresponding to a single peptide concentration fit best using a two-state binding model that indicates an initial weak binding state followed by a conformational change to obtain a strong binding state. For each peptide that showed a detectable binding response, a log-plot of concentration (x-axis) vs relative response (y-axis) based on the individual fits was generated and a single dissociation constant (KD) was computed using the Biacore™ Insight Software based on the two-state model (Table 4).

E.6 Designing Nanobodies that Bind Penguinpox and Hemagglutinin

Experiments by Jacob A. Hambalek, Anshika Gupta, Diego Taquiri Diaz, and Chang C. Liu.

Each nanobody design was cloned into plasmid pMAA28 Hendel et al. [2025], which is a CEN/ARS plasmid that encodes the nanobody as an N-terminal fusion to Aga2 (i.e., N-nanobody-HA-tag-Aga2-C) for display under the control of a pER promoter Yang et al. [2023]. Each plasmid was transformed

bioRxiv preprint doi: https://doi.org/10.1101/2025.11.20.689494; this version posted November 24, 2025. The copyright holder for this preprint (which was not certified by peer review) is the author/funder, who has granted bioRxiv a license to display the preprint in perpetuity. It is made available under aCC-BY 4.0 International license.

into yeast strain yAP174 Wong et al. [2024] and then plated on synthetic complete media lacking histidine, uracil, and leucine (SC-HLU). Colonies for each design were picked into SC-HLU media and grown separately for 18–20 hours at 30°C with 200 rpm. Expression of the surface protein Aga1 was induced with 200 nM β-estradiol, eliciting surface display of the constitutively expressed nanobody–HA tag–Aga2 fusion.

For designs targeting cGAMP PDE, the nanobody-expressing cells were incubated with the labeled cGAMP PDE and an Alexa Fluor 488 conjugated antibody (R&D Systems, catalog #IC6875G), which targets the nanobody's HA tag, in the incubation buffer HBSBM (20 mM HEPES, pH 7.5; 100 mM NaCl; 1 g/L BSA; 1.8 g/L maltose) for 1 hour at 4°C. The cGAMP PDE protein (a gift from Philip J. Kranzusch and Samuel J. Hobbs, Harvard Medical School) was labeled with a reporter dye Alexa Fluor 647 using an NHS-AlexaFluor 647 labeling kit (Thermo Fisher).

For the FhaB-targeting designs, the nanobody-expressing cells were incubated with the FhaB protein for 1 hour at 4°C, followed by incubation with fluorescently labeled Anti-His Alexa Fluor 647 antibody (R&D Systems; catalog #IC0501R) and Anti-HA Alexa Fluor 488 for 30 minutes at 4°C. The FhaB protein (a gift from Celia W. Goulding and Christine D. Hardy, UC Irvine) contains a His-tag for protein purification and detection. After antigen incubation and reporter incubation, 2.5 µg propidium iodide (Sigma-Aldrich; catalog #81845) was added to stain dead cells. The cells were then washed with two volumes of HBSBM and resuspended in 125 µL HBSBM. Each cell population was interrogated for fluorescence using the Attune NxT Flow Cytometer (Thermo Fisher).

E.7 Designing Proteins that Bind to Small Molecules

Experiments by A. Katherine Hatstat, Angelika Arada, Nam Hyeong Kim, Ethel Tackie-Yarboi, Dylan Boselli, Lee Schnaider, and William F. DeGrado

Rational inspection The top 100 computationally ranked designs were examined based on the number and geometry of potential hydrogen bonds formed between rucaparib and each designed binder. rucaparib was conceptually fragmented into three hydrogen-bonding functional groups: carboxamide, indole NH, and secondary amine. Hydrogen bonds were defined by the distance between oxygen or nitrogen atoms of these rucaparib fragments and those of the binder residues within 3.2 Å. The presence of hydrogen bonds involving the carboxamide group was given the highest priority during selection. Six candidate designs were subsequently chosen by visual inspection, considering both burial within the binding pocket and diversity of the protein scaffolds.

Protein expression and purification Codon-optimized genes encoding the designed candidate proteins with an N-terminal 6×His tag and a TEV protease cleavage site (HHHHHHENLYFQS) were synthesized and obtained from Twist Bioscience. To facilitate Gibson assembly into the pET-28a(+) vector, short sequences were added at the 5' end (CTCTAGAAATAATTTTGTTTAACTTTAA-GAAGGAGATATACC) and 3' end (GATCCGGCTGCTAACAAAGCCCGAAAG) of each gene. The recombinant plasmids were transformed into Escherichia coli strain E. cloni BL21(DE3). A single colony was picked from an LB agar plate and inoculated into LB medium supplemented with kanamycin (50 μg/mL) for overnight growth. The culture was then transferred into 200 mL of TB medium containing kanamycin (50 μg/mL) and incubated at 37 °C until reaching an OD600 of 0.6 - 0.8. Protein expression was induced with 0.5 mM isopropyl β-D-1-thiogalactopyranoside (IPTG), and cultures were incubated overnight at 30 °C. Cells were harvested by centrifugation and resuspended in 25 mL PBS buffer (10 mM Na2HPO4, 1.8 mM KH2PO4, 2.7 mM KCl, 137 mM NaCl, pH 7.4) supplemented with 20 mM imidazole. Cells were lysed by ultrasonication (Sonic Dismembrator Model 500, Fisher Scientific), and the lysate was clarified by centrifugation (35,000 g, 30 min). The supernatant was loaded onto a gravity column containing Ni-NTA agarose resin (HisPur, Thermo Fisher, 1.0 mL or 3.0 mL). The resin was washed with three column volumes (CVs) of PBS buffer containing 20 mM imidazole, and bound proteins were eluted with 7 mL PBS buffer containing 250 mM imidazole. The eluted proteins were concentrated and subjected to three rounds of buffer exchange with PBS buffer using a 15 mL, 10 kDa cutoff centrifugal filter unit (EMD Millipore).

Fluorescence emission and fluorescence polarization assays: Fluorescence emission and fluorescence polarization spectra for assessment of rucarparib binding: To assess rucaparib binding, rucaparib

bioRxiv preprint doi: https://doi.org/10.1101/2025.11.20.689494; this version posted November 24, 2025. The copyright holder for this preprint (which was not certified by peer review) is the author/funder, who has granted bioRxiv a license to display the preprint in perpetuity. It is made available under aCC-BY 4.0 International license.

dissolved in DMSO was mixed with proteins in PBS buffer (137 mM NaCl, 2.7 mM KCl, 10 mM Na2HPO4, 1.8 mM KH2PO4, pH 7.4) to a final DMSO concentration below 2%, and incubated for 5 min prior to measurement. Fluorescence emission spectra were recorded in black, flat-bottom 96-well plates using a BioTek Synergy Neo-2 plate reader with an excitation wavelength of 355 nm. Protein aliquots from 10 or 100 μM stocks in PBS were combined to make 200 μL samples containing 10 μM rucaparib. Each condition was measured in triplicate. Fluorescence polarization (FP) assays were performed using the same samples on a BioTek Synergy 2 plate readerequipped with excitation and emission filters of 405 nm and 516 nm, respectively. FP values were recorded in polarization (P) units. The polarization values were plotted against protein concentration, and the data were fitted to a one-site binding model using nonlinear regression in GraphPad Prism 10 to determine the dissociation constant (K_d).

E.8 Designing Antimicrobial Peptides that Inhibit the GyrA to GyrA Interaction

Experiments by Andrew Savinov, and Gene-Wei Li

A library of DNA templates encoding designed variants, mutated variants with 3 alanine substitutions at the binding interface, alongside a library encoding protein fragments tiling GyrA and eGFP, was generated (Twist Biosciences), and massively parallel relative growth measurements in E. coli were performed as previously [Savinov et al., 2022, 2025].

Specifically, the library of coding sequences was cloned into the pET-9a expression vector (Novagen) exactly as previously [Savinov et al., 2022, 2025]. The plasmid library encoding designed binders and protein fragments was then transformed into electrocompetent *E. coli* BL21 (DE3) (Sigma-Aldritch) at ≥110-fold coverage of the library size, and following 1-hr recovery from transformation, cells were immediately diluted into LB media (Gibco) containing kananycin (selecting for presence of the library) and 10 uM IPTG (inducer for library expression), beginning the massively parallel inhibition measurements. Cells were then grown to an OD_{600nm} of 1.5, at which point they were harvested. These experiments were performed in triplicate (3 biological replicates). Plasmids were extracted from each sample (Qiagen miniprep kit), and DNA from each output sample as well as the plasmid library input was prepared for high-throughput sequencing as previously [Savinov et al., 2022]. Paired-end sequencing was performed on a Singular G4 platform. From these measurements we determined designed peptide and protein fragment frequencies in the population (f) at the initial and final growth assay timepoints, allowing calculation of the enrichment E = log2(f_initial/f_final). The inhibition score for each peptide was then calculated as Inhibition = -E, such that larger positive values correspond to stronger inhibitory effects. Results across biological replicates of these measurements were highly reproducible as in prior work [Savinov et al., 2022, 2025].

The specificity of designed binders for the designed binding mode to GyrA was calculated as Δ(Inhibition) = Inhibition(designed binder) - Inhibition(mutated binder). Positive Δ(Inhibition) values therefore correspond to binders which are more inhibitory than their corresponding variants where 3 interface residues are mutated to alanine. Designed binders and protein fragments that substantially inhibit cell growth were defined as those with inhibition scores $E \leq$ -2, corresponding to a ≥4-fold reduction in relative growth. Results were similar with a less stringent threshold of $E \leq$ -1, and so the more conservative threshold was employed. This threshold picks out previously identified inhibitory peaks from protein fragments tiling across GyrA [Savinov et al., 2022, 2025].
